# Elucidating the role of the gut microbiota in the physiological effects of dietary fiber

**DOI:** 10.1186/s40168-022-01248-5

**Published:** 2022-05-13

**Authors:** Edward C. Deehan, Zhengxiao Zhang, Alessandra Riva, Anissa M. Armet, Maria Elisa Perez-Muñoz, Nguyen K. Nguyen, Jacqueline A. Krysa, Benjamin Seethaler, Yuan-Yuan Zhao, Janis Cole, Fuyong Li, Bela Hausmann, Andreas Spittler, Julie-Anne Nazare, Nathalie M. Delzenne, Jonathan M. Curtis, Wendy V. Wismer, Spencer D. Proctor, Jeffrey A. Bakal, Stephan C. Bischoff, Dan Knights, Catherine J. Field, David Berry, Carla M. Prado, Jens Walter

**Affiliations:** 1 Department of Agricultural, Food and Nutritional Science, University of Alberta, Edmonton, Alberta Canada; 2 Department of Medicine, University of Alberta, Edmonton, Alberta Canada; 3 College of Food and Biological Engineering, Jimei University, Xiamen, Fujian China; 4 Department of Microbiology and Ecosystem Science, Division of Microbial Ecology, Centre for Microbiology and Environmental Systems Science, University of Vienna, Vienna, Austria; 5 Metabolic and Cardiovascular Disease Laboratory, University of Alberta, Edmonton, Alberta Canada; 6 Institute of Nutritional Medicine, University of Hohenheim, Stuttgart, Germany; 7 Joint Microbiome Facility of the Medical University of Vienna and University of Vienna, Vienna, Austria; 8 Department of Laboratory Medicine, Medical University of Vienna, Vienna, Austria; 9 Core Facility Flow Cytometry and Department of Surgery, Research Lab, Medical University of Vienna, Vienna, Austria; 10 Centre de Recherche en Nutrition Humaine Rhône-Alpes, Univ-Lyon, CarMeN Laboratory, INSERM, INRA, INSA Lyon, Université Claude Bernard Lyon 1, Hospices Civils de Lyon, F-CRIN/FORCE Network, Pierre-Bénite, France; 11 Metabolism and Nutrition Research Group, Louvain Drug Research Institute, Université Catholique de Louvain, Brussels, Belgium; 12 Patient Health Outcomes Research and Clinical Effectiveness Unit, Division of General Internal Medicine, University of Alberta, Edmonton, Alberta Canada; 13 Department of Computer Science and Engineering, University of Minnesota, Minneapolis, Minnesota USA; 14 BioTechnology Institute, University of Minnesota, Saint Paul, Minnesota USA; 15 Department of Biological Sciences, University of Alberta, Edmonton, Alberta Canada; 16 APC Microbiome Ireland, School of Microbiology, and Department of Medicine, University College Cork – National University of Ireland, Cork, Ireland

**Keywords:** Dietary fiber, Adults, Obesity, Satiety, Insulin resistance, Inflammation, Gut microbiota

## Abstract

**Background:**

Dietary fiber is an integral part of a healthy diet, but questions remain about the mechanisms that underlie effects and the causal contributions of the gut microbiota. Here, we performed a 6-week exploratory trial in adults with excess weight (BMI: 25–35 kg/m^2^) to compare the effects of a high-dose (females: 25 g/day; males: 35 g/day) supplement of fermentable corn bran arabinoxylan (AX; *n* = 15) with that of microbiota-non-accessible microcrystalline cellulose (MCC; *n* = 16). Obesity-related surrogate endpoints and biomarkers of host-microbiome interactions implicated in the pathophysiology of obesity (trimethylamine *N*-oxide, gut hormones, cytokines, and measures of intestinal barrier integrity) were assessed. We then determined whether clinical outcomes could be predicted by fecal microbiota features or mechanistic biomarkers.

**Results:**

AX enhanced satiety after a meal and decreased homeostatic model assessment of insulin resistance (HOMA-IR), while MCC reduced tumor necrosis factor-α and fecal calprotectin. Machine learning models determined that effects on satiety could be predicted by fecal bacterial taxa that utilized AX, as identified by bioorthogonal non-canonical amino acid tagging. Reductions in HOMA-IR and calprotectin were associated with shifts in fecal bile acids, but correlations were negative, suggesting that the benefits of fiber may not be mediated by their effects on bile acid pools. Biomarkers of host-microbiome interactions often linked to bacterial metabolites derived from fiber fermentation (short-chain fatty acids) were not affected by AX supplementation when compared to non-accessible MCC.

**Conclusion:**

This study demonstrates the efficacy of purified dietary fibers when used as supplements and suggests that satietogenic effects of AX may be linked to bacterial taxa that ferment the fiber or utilize breakdown products. Other effects are likely microbiome independent. The findings provide a basis for fiber-type specific therapeutic applications and their personalization.

**Trial registration:**

Clinicaltrials.gov, NCT02322112, registered on July 3, 2015.

Video Abstract

**Supplementary Information:**

The online version contains supplementary material available at 10.1186/s40168-022-01248-5.

## Background

Obesity and its comorbidities such as type II diabetes have reached epidemic proportions worldwide [1]. Observational research has linked dietary fiber with reduced prevalence of chronic diseases [2, 3], and mechanisms by which fibers exert their benefits have been established in animal models [4, 5]. The health effects of fibers are dependent on their physicochemical properties [6, 7]. Viscous fibers show efficacy in improving lipid and glucose metabolism [8, 9], which is reflected in a health claim by the European Food Safety Authority for moderately viscous wheat endosperm arabinoxylans (AXs) and postprandial glycemic control [10]. Given that average fiber consumption remains low in industrialized societies [11], fiber supplementation could be an effective treatment or preventive strategy for obesity-related chronic diseases [12].

Clinical evidence for the health effects of fiber supplements remains highly inconsistent [13], and it has been questioned whether purified (isolated) forms of fiber maintain their physiological effects once removed from the three-dimensional plant cell wall matrix [2, 14]. In this context, the mechanistic foundations for the beneficial effects of purified fibers remain insufficiently understood in humans. The strongest evidence exists for viscous fibers, which likely prolong satiety and lower postprandial metabolic responses by delaying gastric emptying and intestinal nutrient absorption [7]. Fermentable fibers are hypothesized to favorably modulate the gut microbiota [4, 15] in a manner that mitigates obesity and related comorbidities [16, 17]. For example, selective fermentation of fibers can alter compositional features of the gut microbiota in a structure-dependent manner [18, 19], selecting for bacterial taxa associated with metabolic effects (e.g., *Prevotella copri* [20] and *Eubacterium rectale* [21]). Moreover, fiber fermentation generates metabolites such as short-chain fatty acids (SCFAs), which act as signaling molecules that may maintain intestinal barrier integrity and immune homeostasis, and induce hormones that regulate satiety and glucose metabolism [5, 22]. Some fiber structures can also bind bile acids, which display immunomodulatory and metabolic properties [23, 24]. The reconfiguration of the bile acid pool might, therefore, constitute another mechanism for the physiological effects of fiber [15].

Although the role of the gut microbiome in the health effects of dietary fiber has received tremendous attention [15, 25], studies in humans have so far only established correlations between physiological effects and shifts in specific bacterial taxa [26, 27] or metabolites [28, 29] that cannot assign causality [30]. A causal role of the microbiome has been established in animal models [31], but experiments in conventional and germ-free mice have demonstrated that the effects of fiber can also be completely independent of the gut microbiota [32]. Therefore, it remains unclear whether the gut microbiota is mechanistically implicated in the physiological effects of fiber, and if so, which effects are microbiome-dependent and which microbes are involved. Although causality is difficult to establish in humans [30], comparisons of physicochemically distinct fibers that differ in their degree of fermentability could be used to determine whether health outcomes are predictable through compositional and functional responses of the gut microbiota.

In a previous study [33], we compared the effects of high doses of two purified fibers—a moderately viscous [34] and fermentable [35] AX with an insoluble, non-accessible [33] large-particle microcrystalline cellulose (MCC)—on compositional and functional features of fecal microbiota in adults with excess weight (body mass index [BMI]: 25–35 kg/m^2^). This study showed that AX, but not MCC, induced global shifts in the fecal microbiota, and enriched, although with a substantial degree of inter-individual variation, bacterial taxa (e.g., *P. copri*) [20, 36] and metabolic functions (propionate) [36–38] implicated in host metabolic features linked to obesity and satiety. Here, we extended this research and assessed the effects of the purified fibers on perceived satiety and obesity-related surrogate endpoints in the same individuals. We hypothesized that administration of a fermentable AX would induce physiological effects that were linked to compositional and functional changes in fecal microbiota (e.g., fermentation), as well as molecular markers of biological processes induced through microbiome metabolites (e.g., SCFAs). To test this hypothesis, we compared the physiological effects of AX with non-accessible MCC and explored associations with fecal microbiota compositional (fiber-responsive taxa and ecological variables of the broader community [33]) and functional (SCFAs and bile acids) features. To gain insight into potential mechanisms, we assessed biomarkers of host-microbiome interactions implicated in the pathophysiology of obesity (i.e., trimethylamine *N*-oxide [TMAO], gut hormones, cytokines, and measures of intestinal barrier integrity). To determine the role of fiber fermentation, we analyzed the fecal microbiota from participants that consumed AX to identify bacterial taxa that were involved in the utilization of AX by employing an approach that combined ex vivo anaerobic fermentations with bioorthogonal non-canonical amino acid tagging (BONCAT) and fluorescence-activated cell sorting (FACS) [39]. We integrated the data using a machine learning approach to determine whether effects on satiety or surrogate endpoints could be predicted by fecal microbiota features or biomarkers of host-microbiome interactions.

## Results

### Baseline characteristics of study participants

**Fig. 1:**
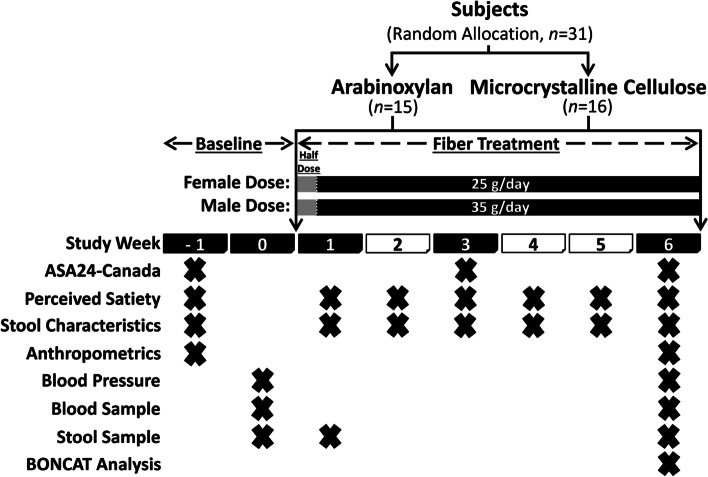
Study design of the randomized controlled trial. ASA24-Canada, Canadian version of the web-based Automated Self-Administered 24-hour Dietary Assessment Tool; stool characteristics, self-reported stool consistency and bowel movement frequency

2To compare the effects of AX and MCC supplements at high doses (females: 25 g/day; males: 35 g/day) and high purity (> 80% fiber) on human health, we performed a single-blind, parallel-arm, 6-week, randomized controlled exploratory trial in adults with excess weight (Fig. 1). A total of 31 participants (AX: 10F and 5M; MCC: 11F and 5M) aged 32.9 ± 8.5 years with a BMI of 28.7 ± 2.3 kg/m^2^ completed the study protocol and were included in statistical analyses. Mean protocol adherence to AX and MCC supplementation was 94.7 ± 6.5% and 95.0 ± 5.6%, respectively (Additional file 1: Figure S1). No differences in age, sex, surrogate endpoints, or other study variables were detected between groups at baseline (see Additional file 2: Table S1 for baseline characteristics).

### Dietary intake

Dietary fiber intake increased by 142% and 171% during fiber supplementation of AX and MCC, respectively, as compared to baseline (*p* = 0.0002 for both AX and MCC, permutational *t*-test) with no difference between groups. This corresponded to an increase from 21 ± 6 and 19 ± 8 g/day to 46 ± 12 and 44 ± 8 g/day for AX and MCC, respectively (Additional file 3: Table S2). Interestingly, sugar consumption also increased by 35% during AX (*p* = 0.04) and 46% during MCC (*p* = 0.03) supplementation, likely due to participants incorporating the powdered supplements into foods and drinks that contained sugar, such as yogurt and fruit smoothies. No differences were detected between groups (*p* > 0.1), suggesting similar dietary changes were made by participants in both groups.

### AX and MCC differ markedly in their physiological effects

**Fig. 2:**
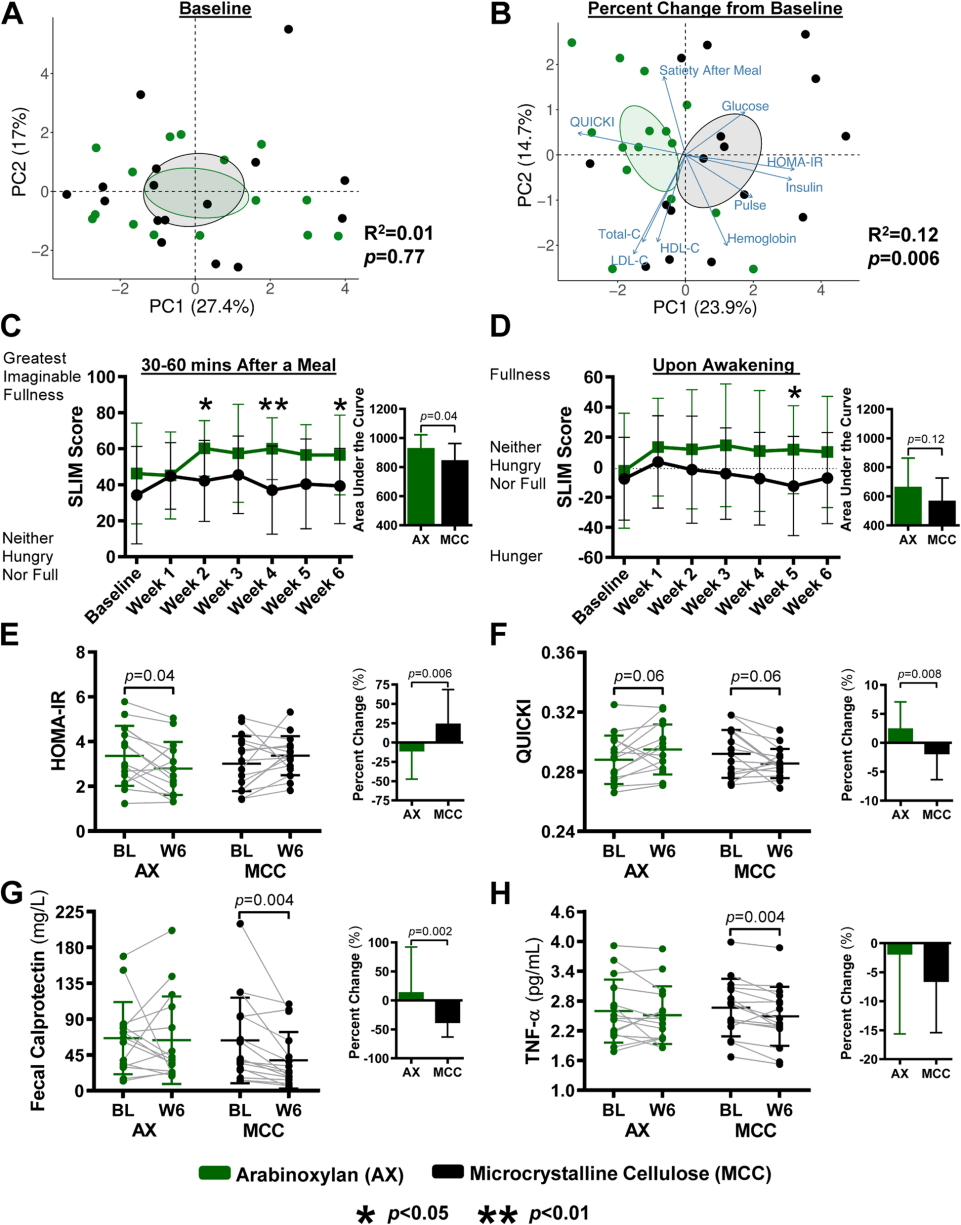
Effects of AX and MCC supplementation on satiety and surrogate endpoints. Principal component analysis plots show **A** perceived satiety and surrogate endpoints at baseline and **B** their percentage change from baseline per AX and MCC groups. Line graphs show weekly SLIM scale ratings **C** 30–60 min after consuming a meal with AX or MCC and **D** upon awakening. Bars (insets) represent the area under the SLIM score curve (AUC_BL–W6_). Scatter plots show **E** HOMA-IR, **F** QUICKI, **G** fecal calprotectin, and **H** TNF-α at baseline and week 6 of AX or MCC supplementation, respectively. Bars (insets) represent the percent change from baseline values per group. To assess within-group changes relative to baseline, data were analyzed for **C** and **D** using repeated measures one-way ANOVA with permutations and for **E** to **H** using paired permutational *t*-tests. To assess between-group differences, data were analyzed for **A** and **B** using permutational multivariate analysis of variance based on Manhattan distance and for **C** to **H** using unpaired permutational *t*-tests. Statistical significance was set for **A** to **D** at *p* < 0.05 and for **E** to **H** at *p* < 0.01. Data for **C** to **H** presented as mean ± SD; for **E** to **H** symbols represent individual samples. AX, arabinoxylan; HOMA-IR, homeostatic model assessment of insulin resistance; MCC, microcrystalline cellulose; QUICKI, quantitative insulin sensitivity check index; SLIM, Satiety Labeled Intensity Magnitude; TNF-α, tumor necrosis factor-α

Principal component analysis ordination of perceived satiety and surrogate endpoints revealed no differences at baseline between the AX and MCC groups (*p* = 0.77, permutational multivariate analysis of variance; Fig. 2A). In contrast, shifts in the variables from baseline to week 6 showed strong clustering by treatment group (*p* = 0.006; Fig. 2B), indicating that the two fibers differed in their overall physiological effects.

#### Effects of AX and MCC on perceived satiety

AX resulted in higher satiety scores, assessed by the Satiety Labeled Intensity Magnitude (SLIM) scale, 30–60 min after consuming a meal (referred to as ‘satiety after a meal’) compared to MCC (*p* = 0.035, permutational *t*-test), with between-group differences detected during weeks 2 (*p* = 0.04), 4 (*p* = 0.007), and 6 (*p* = 0.03) (Fig. 2C). Further evaluation of satiety scores revealed that after AX consumption, individuals perceived feeling between “moderately full” and “very full”, while those consuming MCC remained between “slightly full” and “moderately full”. After participants awoke, AX also consistently increased satiety scores towards feeling “slightly full” over the entire treatment period, while MCC tended to reduce satiety scores towards feeling “slightly hungry,” with between-group differences reaching significance during week 5 (*p* = 0.03) (Fig. 2D).

#### Effects of AX and MCC on obesity-related surrogate endpoints and biomarkers of host-microbiome interactions

Among the surrogate endpoints, AX consumption reduced homeostatic model assessment of insulin resistance (HOMA-IR; insulin resistance index; *p* = 0.006; Fig. 2E) and increased quantitative insulin sensitivity check index (QUICKI; insulin sensitivity index, *p* = 0.008; Fig. 2F) compared to the MCC group. Effects of AX on HOMA-IR showed a 36% difference relative to MCC, which is comparable to those reported for other microbiome-targeted strategies, such as fecal microbiota transplantation (FMT) [40–42]. MCC reduced fecal calprotectin (a surrogate endpoint of intestinal inflammation) when compared to the AX group (*p* = 0.002), and by 39% relative to baseline (*p* = 0.004) (Fig. 2G). No effects of AX or MCC were detected for any other surrogate endpoint (Additional file 4: Table S3).

Evaluating biomarkers of host-microbiome interactions revealed a 7% reduction from baseline in tumor necrosis factor-α (TNF-α) by MCC (*p* = 0.004; Fig. 2H). No other effects of AX or MCC were detected for the remaining biomarkers of host-microbiome interactions (Additional file 4: Table S3), despite the fact that the production of gut hormones and intestinal barrier integrity have been previously linked to fiber fermentation [5, 16].

To confirm significant effects were independent of potential confounders, analysis of covariance (ANCOVA) models were performed using age, sex, and changes in total fiber and sugar consumption as covariates (Additional file 5: Table S4). Stool characteristic variables were also included since obesity has been associated with altered bowel habits [43] and, in our preceding study [33], AX and MCC promoted more frequent bowel movements (*p* < 0.05), while AX promoted softer stool consistencies compared to MCC (*p* < 0.05) (Additional file 4: Table S3). ANCOVA models showed that the observed effects were not confounded by host factors, dietary changes, or stool characteristics (*p* < 0.05).

#### Effects of AX and MCC on functional features of the fecal microbiota

As previously shown [33], AX consumption directed microbial output of SCFAs in favor of propionate, while MCC did not alter fecal SCFAs. Since bile acid derivatives also possess immunomodulatory and metabolic properties [23, 24], we applied targeted metabolomics to determine the effects of AX and MCC on fecal bile acids. This analysis showed that MCC decreased concentrations of apocholic acid (*p* = 0.009, permutational *t*-test) and hyodeoxycholic acid (*p* = 0.009) relative to baseline. Reductions in total fecal concentrations of bile acids and five secondary bile acids—deoxycholic acid, isolithocholic acid (ILCA), taurolithocholic acid (TLCA), taurodeoxycholic acid (TDCA), and glycodeoxycholic acid (GDCA)—also approached significance (0.01 < *p <* 0.05; Table 1 and Additional file 6: Table S5). By contrast, AX did not reduce bile acid concentrations relative to baseline but, when compared to MCC, increased concentrations of 7αOH-3-oxo-4-cholestenoic acid (*p* = 0.0096). Changes induced by both treatments also showed large standard deviations, indicating that bile acid shifts were highly individualized. Overall, our findings suggest that consumption of large-particle MCC alters the fecal bile acid profile by reducing secondary bile acid concentrations.

**Table 1:** Fecal Concentrations of the most prevalent bile acids detected at baseline and 6 weeks of arabinoxylan or microcrystalline cellulose supplementation

Fecal bile acids (nmol/g)	Arabinoxylan (*n* = 15)				Microcrystalline cellulose (*n* = 16)				Between group change *p* value
	Baseline	Week 6	Within group *p* value	Change (W6–BL)	Baseline	Week 6	Within group *p* value	Change (W6–BL)	
**Total bile acids**	90140.7 ± 52067.3	75570.8 ± 36138.7	0.13	− 14569.9 ± 35493.8	76748.0 ± 45152.6	53400.4 ± 42156.7	**0.03**	− 23347.6 ± 41145.7	0.88
**Primary bile acids**	4240.6 ± 11609.1 ^a^	1518.5 ± 3629.2 ^a^	0.48	− 2722.1 ± 10503.0 ^a^	214.0 ± 278.3	1457.7 ± 3939.2	0.46	1243.7 ± 3948.3	0.20
Cholic acid	1289.4 ± 4663.3 ^a^	623.9 ± 1541.4 ^a^	0.77	− 665.5 ± 4094.5 ^a^	49.9 ± 79.3	781.0 ± 2150.8	0.45	731.1 ± 2154.9	0.64
Chenodeoxycholic acid	1392.3 ± 2752.4 ^a^	2339.4 ± 6773.5 ^a^	0.70	947.1 ± 6328.0 ^a^	78.1 ± 96.4	640.2 ± 1739.7	0.46	562.1 ± 1748.3	1.00
Glycocholic acid	16.2 ± 13.9	17.3 ± 13.8	0.78	1.1 ± 15.6	15.9 ± 18.8 ^a^	9.0 ± 7.1 ^a^	0.07	− 6.8 ± 14.6 ^a^	0.18
Glycochenodeoxycholic acid	16.3 ± 14.7 ^a^	16.9 ± 12.3 ^a^	0.90	0.6 ± 15.6 ^a^	26.8 ± 32.9	13.1 ± 14.6	0.11	− 13.6 ± 34.2	0.15
Taurocholic acid	6.5 ± 6.0	15.5 ± 37.4	0.61	9.0 ± 38.1	5.4 ± 5.7 ^a^	8.5 ± 22.4 ^a^	0.92	3.1 ± 21.2 ^a^	0.59
Taurochenodexycholic acid	11.9 ± 27.2	7.6 ± 13.5	0.69	− 4.3 ± 30.7	7.8 ± 10.1 ^a^	6.7 ± 18.2 ^a^	0.88	− 1.1 ± 21.0 ^a^	0.76
**Secondary bile acids**	85635.9 ± 48491.8	69467.5 ± 35335.1	0.12	− 16168.4 ± 37401.2	76533.0 ± 45107.0	51941.9 ± 42028.7	**0.03**	− 24591.1 ± 41860.1	0.60
Lithocholic acid	32898.7 ± 20028.4	24456.2 ± 14586.5	0.06	− 8442.5 ± 15869.9	31183.8 ± 15926.9	21373.9 ± 16591.2	0.06	− 9809.9 ± 18874.8	0.92
Deoxycholic acid	46951.5 ± 31821.9	39324.1 ± 24346.5	0.28	− 7627.4 ± 26367.7	38305.5 ± 24802.5	25727.3 ± ± 21327.7	**0.04**	− 12578.2 ± 23022.2	0.88
Glycolithocholic acid	2.4 ± 2.1	1.6 ± 1.3	0.30	− 0.8 ± 2.7	2.0 ± 1.6	1.4 ± 1.1	0.16	− 0.7 ± 1.8	1.00
Glycodeoxycholic acid	18.5 ± 14.8	15.0 ± 11.1	0.45	− 3.5 ± 17.8	24.1 ± 24.4 ^a^	8.3 ± 6.1 ^a^	**0.014**	− 15.8 ± 24.4 ^a^	0.20
Taurolithocholic acid	0.8 ± 1.1	2.1 ± 4.6	0.20	1.3 ± 4.3	3.3 ± 5.9	1.1 ± 2.8	**0.03**	− 2.2 ± 4.0	**0.018**
Allocholic acid	58.6 ± 81.7	56.5 ± 79.8	0.93	− 2.2 ± 106.5	21.7 ± 26.2	18.5 ± 26.0	0.58	− 3.2 ± 22.6	1.00
Apocholic acid	742.1 ± 681.7	493.9 ± 246.7	0.15	− 248.2 ± 626.0	836.2 ± 1074.2 ^a^	318.4 ± 177.8 ^a^	0.009 *	− 517.8 ± 1009.5 ^a^	0.88
Alloisolithocholic acid	124.0 ± 103.4	110.0 ± 90.0	0.43	− 14.0 ± 65.7	187.2 ± 140.3 ^a^	118.3 ± 99.5 ^a^	0.07	− 68.9 ± 135.2 ^a^	0.26
Isolithocholic acid	1867.0 ± 1020.8	1524.4 ± 987.1	0.28	− 342.6 ± 1183.0	3133.7 ± 2649.2	1669.6 ± 2070.6	**0.016**	− 1464.1 ± 2298.5	0.11
Hyodeoxycholic acid	145.7 ± 120.9	136.7 ± 119.8	0.80	− 9.1 ± 133.9	207.6 ± 192.2 ^a^	96.0 ± 81.5 ^a^	0.009 *	− 111.6 ± 185.4 ^a^	0.08
Dehydrolithocholic acid	215.6 ± 123.4	173.1 ± 108.2	0.16	− 42.5 ± 112.5	425.7 ± 759.4 ^a^	187.1 ± 171.9 ^a^	0.05	-238.6 ± 636.0 ^a^	0.26
Ursocholic acid	295.3 ± 642.2	307.1 ± 849.4	0.96	11.8 ± 1074.5	30.0 ± 51.2	259.7 ± 800.7	0.26	229.7 ± 804.7	0.66
Murocholic acid	18.8 ± 19.8	12.1 ± 10.8	0.18	− 6.7 ± 18.7	13.5 ± 15.7	7.4 ± 6.4	0.07	− 6.1 ± 12.9	0.92
Norcholic acid	9.5 ± 4.3	8.4 ± 4.7	0.23	− 1.1 ± 3.4	8.0 ± 5.1	6.2 ± 5.5	0.15	− 1.8 ± 4.8	0.39
Nordeoxycholic acid	1.7 ± 1.2	1.4 ± 1.2	0.18	− 0.3 ± 0.8	2.5 ± 2.1 ^a^	1.6 ± 1.4 ^a^	0.06	− 0.8 ± 1.1 ^a^	0.11
7-Ketodeoxycholic acid	23.7 ± 67.6 ^a^	19.3 ± 32.4 ^a^	0.88	− 4.4 ± 57.1 ^a^	5.1 ± 4.4	58.1 ± 188.1	0.45	53.0 ± 188.0	0.23
7-Ketolithocholic acid	144.8 ± 422.4 ^a^	30.7 ± 35.8 ^a^	0.27	− 114.1 ± 393.6 ^a^	11.0 ± 9.7	31.1 ± 79.6	0.46	20.2 ± 80.8	**0.047**
12-Ketochenodeoxycholic acid	9.7 ± 4.1	8.4 ± 5.5	**0.03**	− 1.4 ± 2.4	7.3 ± 3.1	6.7 ± 3.5	0.61	− 0.6 ± 4.2	0.75
3βOH-5-cholestenoic acid	227.1 ± 154.4	225.5 ± 184.5	0.96	− 1.6 ± 145.9	120.5 ± 82.4	86.9 ± 33.3	0.07	− 33.6 ± 67.9	0.84
7αOH-3-oxo-4-cholestenoic acid	1.7 ± 1.3 ^a^	4.0 ± 4.0 ^a^	**0.04**	2.3 ± 4.3 ^a^	1.6 ± 1.3	1.4 ± 1.6	0.39	− 0.2 ± 0.9	0.0096 *
Cholic acid 3-SO_4_^2−^	65.5 ± 172.7	38.9 ± 147.1	0.96	− 26.6 ± 237.8	74.5 ± 202.6	0.8 ± 1.0	0.39	− 73.7 ± 202.4	0.90
Lithocholic acid 3-SO_4_^2−^	113.9 ± 263.0	83.1 ± 257.5	0.59	− 30.8 ± 275.2	34.4 ± 65.8 ^a^	98.3 ± 301.0 ^a^	0.39	63.9 ± 315.5 ^a^	0.54
Deoxycholic acid 3-SO_4_^2−^	33.7 ± 96.7 ^a^	616.9 ± 2293.9 ^a^	0.78	583.3 ± 2290.2 ^a^	274.3 ± 745.5	128.6 ± 428.1	0.39	− 145.7 ± 857.7	0.19
Glycolithocholic acid 3-SO_4_^2−^	5.0 ± 8.2	2.6 ± 1.3	0.27	− 2.4 ± 8.1	6.7 ± 8.2 ^a^	4.4 ± 8.0 ^a^	0.39	− 2.2 ± 12.1 ^a^	0.94
Glycodeoxycholic acid 3-SO_4_^2−^	1.0 ± 1.0	0.6 ± 0.5	0.20	− 0.4 ± 1.1	1.2 ± 1.6 ^a^	0.4 ± 0.4 ^a^	0.39	− 0.8 ± 1.5 ^a^	0.76

Listed bile acids (31 compounds) were detected in > 90% of fecal samples. Statistical significance of within-group shifts were determined by paired permutational *t*-tests, while between-group differences (AX vs MCC; W6–BL, week 6–baseline) were determined by unpaired permutational *t*-tests. Data are means ± SD. * Statistical significance was set at *p* < 0.01, bolded *p* values without an asterisk (*) are approaching statistical significance (*p* < 0.05). ^a^ One outlier > 5*SD from the mean was excluded

### Identification of bacterial consortia involved in AX degradation

**Fig. 3:**
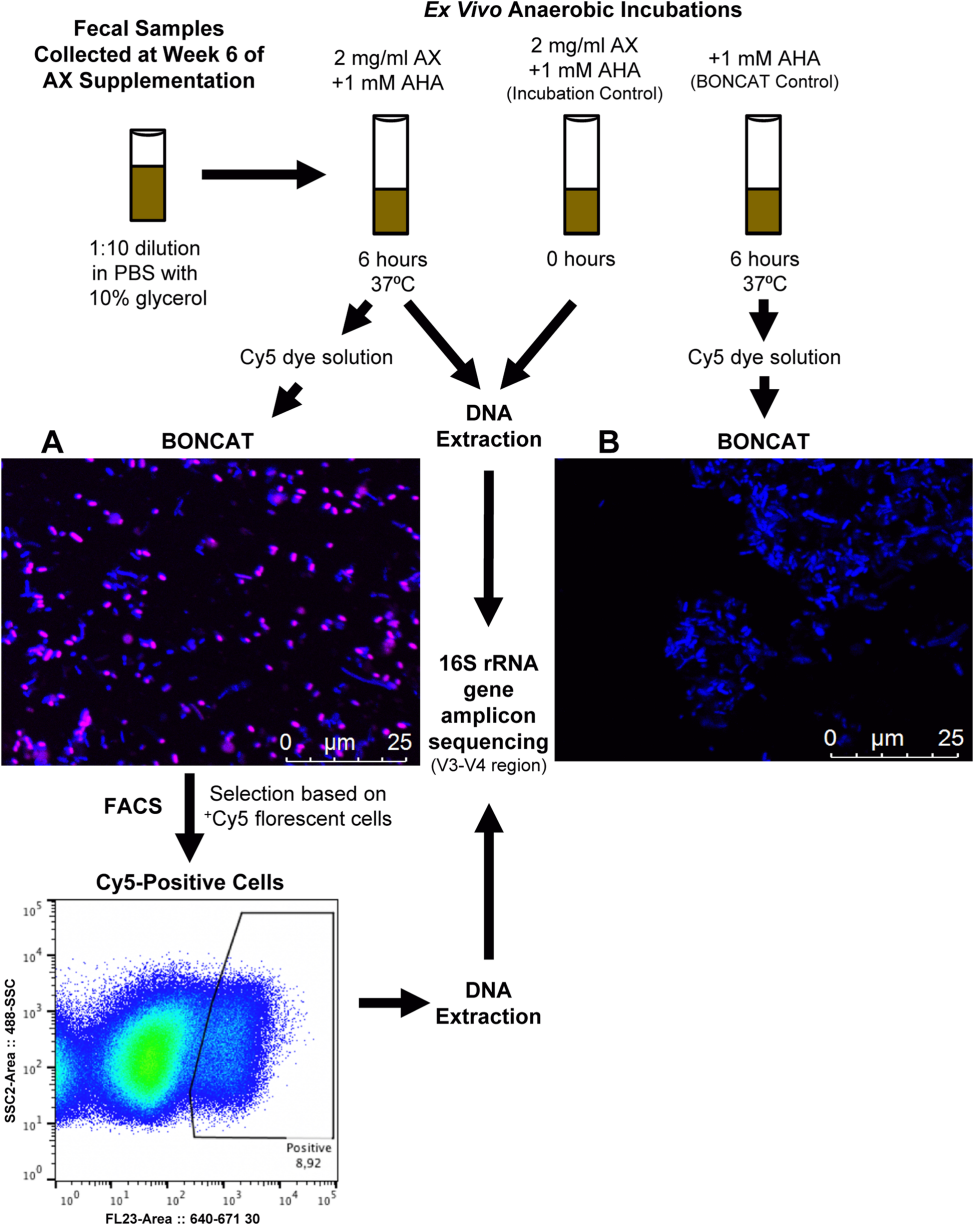
Schematic representation of the ex vivo detection assay based on BONCAT. Stool samples stored frozen were thawed, filtered, and washed in PBS and then incubated in the presence of AX and the cellular activity marker *L*-azidohomoalanine (AHA) to detect AX-stimulated bacterial cells. A no-amendment control, containing only AHA, was incubated to detect possible basal activity in the absence of AX. Microscopic inspection showed no BONCAT signal for all controls; thus, no basal activity was detected. AX-incubated samples were then fixed in ethanol and active cells were stained using a Cu(I)-catalyzed click reaction using a Cy5 dye solution. **A** and **B** A representative picture of fecal microbiota incubated for 6 h **A** with AX and **B** without AX (BONCAT control). Stimulated cells, shown in pink as a Cy5-positive BONCAT signal, were sorted by FACS, with all microbial cells shown in blue (DAPI stained). DNA was extracted from both sorted cells and samples at 0-h and 6-h anaerobic incubations. The 16S rRNA gene was amplified by PCR and amplicons were sequenced using the Illumina Miseq platform. AX, arabinoxylan; BONCAT, bioorthogonal non-canonical amino acid tagging; FACS, fluorescence-activated cell sorting

Considering that bacteria involved in fiber fermentation dictate the production of health-related metabolites [44], we aimed to identify the bacterial taxa within participants’ fecal microbiota that were involved in the fermentation of AX and utilization of breakdown products released during fermentation. We used BONCAT to fluorescently label metabolically active bacterial cells within the fecal microbiota [39], then sorted and profiled the active cells by FACS and 16S rRNA gene amplicon sequencing, respectively (Fig. 3 and Additional file 7: Figure S2). Initial comparison of fluorescent cells in incubations that contained the cellular activity marker *L*-azidohomoalanine (AHA) plus Cy5-dye, but differed by AX amendment, revealed no basal cellular activity in the absence of AX; thus, demonstrating that BONCAT was highly specific and only detected AX-induced cellular activity (Fig. 3).

Compared to the total bacterial community prior to incubation with AX, the active consortia had lower α-diversity (Shannon index: *p* = 0.0008, one-way ANOVA with permutations) and richness (Chao1 index: *p* < 0.0001) with, on average, 31% fewer amplicon sequence variant (ASV) numbers (*p* = 0.0001) (Additional file 8: Table S6). Several bacterial taxa previously shown to utilize AX were represented among the 31 dominant ASVs (mean relative abundance > 1%) in the active consortia (Additional file 8: Table S6), including ASVs related to *Bifidobacterium longum*, *Blautia obeum*, *Bacteroides ovatus*, *Bacteroides cellulosilyticus*, and *E. rectale* [33, 45]. Overall, these findings suggest that AX fermentation is not limited to a few cooperative species but extends to numerous members of the broader bacterial community, and involves several primary degraders as well as secondary fermenters.

To compare the abundance of individual taxa between the active consortia and fecal bacterial community, a differential abundance test (DESeq2) was applied. This analysis revealed the families *Bacteroidaceae*, *Lactobacillaceae,* and *Enterobacteriaceae* were more abundant in the active consortia, while *Rikenellaceae*, *Ruminococcaceae*, and *Streptococcaceae* were underrepresented. Fourteen ASVs were identified to differ between the active consortia and fecal bacterial communities prior to incubation with AX. *Bacteroides koreensis*, *Bacteroides plebeius*, *Bacteroides xylanisolvens*, *Lactobacillus* spp., and *Escherichia/Shigella* spp. were more abundant in the active consortia (*p* < 0.01). ASVs related to *Coprococcus eutactus*, *Faecalibacterium prausnitzii*, and *Dialister invisus* [46], which might be utilizing sugars and metabolic by-products (e.g., acetate and lactate) released during AX degradation [5, 33], were also metabolically active during incubation with AX but less abundant in the active consortia (*p* < 0.01).

### Identification of microbiota-related predictors of satiety and surrogate endpoints

To gain insight into the role of the gut microbiota in the physiological effects of fiber, we used a machine learning approach to determine predictors for the physiological effects of the fibers on perceived satiety and surrogate endpoints. Variables related to microbiota compositional (fiber-responsive taxa in feces [33], BONCAT identified active taxa [differentially abundant bacterial ASVs and all metabolically active bacterial ASVs with average relative abundances ≥ 0.15%] and ecological characteristics [α-diversity and principal components] [33]) and functional (SCFAs and bile acids) features, biomarkers of host-microbiome interactions (TMAO, gut hormone, cytokine, and barrier function measures), and calorie-adjusted macronutrient intake data were included in the models. For each endpoint affected by fiber consumption, high- and low-responders were first identified according to the study cohort median (Additional file 9: Figures S3A and S3B), and datasets were used as predictor variables for the training of independent random forest classifiers (RFCs) to rank microbiota-related predictors that discriminate high-responders from low-responders (Additional file 9: Figures S3C and S3D).

**Fig. 4:**
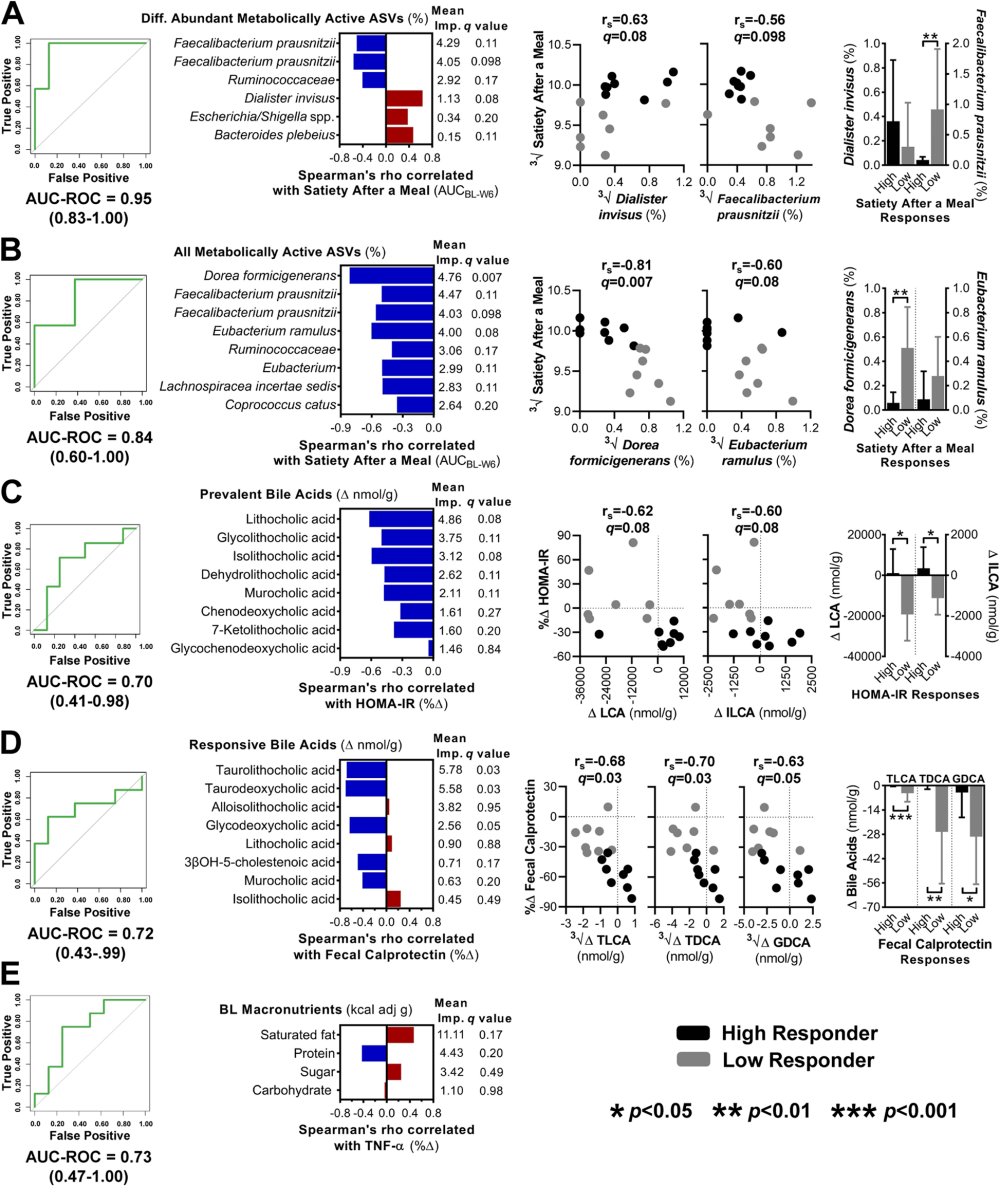
Identification of gut microbiota compositional features and biomarkers of host-microbiome interactions that predict satiety and surrogate endpoint responses by machine learning. (left) AUC-ROC curves show the performance accuracy of random forest classifiers trained to predict high-vs-low responders for: **A** and **B** perceived satiety after a meal with AX using the relative abundance of bacterial taxa activated during ex vivo incubation with AX; **C** HOMA-IR and **D** fecal calprotectin for AX and MCC, respectively, using fecal bile acid shifts; and **E** TNF-α for MCC using baseline intakes of calorie-adjusted macronutrients. (center) Horizontal bars represent Spearman’s correlation coefficients between endpoints and **A **and **B **metabolically active ASVs, **C** and **D** fecal bile acids, or **E** macronutrients shown to be important for predicting responses. Mean importance values were determined by random forest, which identifies factors that contribute the most to the model. (right) Scatter plots show the association between endpoints and the most discriminative microbiota-related factors that correlate with AX-induced **A** and **B** satiety after a meal and **C** HOMA-IR attenuation, and **D** MCC-induced fecal calprotectin attenuation. Vertical bar graphs show the most discriminative microbiota-related factors grouped by high- and low-responders. High-responders (black) and low-responders (gray) were defined according to the study cohort median. Statistical significance was set at *p* < 0.05 and FDR adjusted *q* values < 0.05. ∆, absolute change from baseline to week 6; %∆, percent change from baseline to week 6; ^3^√, cube root transformed before analysis; All ASVs, amplicon sequence variants with average relative abundances ≥ 0.15%; AX, arabinoxylan; AUC-ROC, area under the receiver operating characteristic curve; BL, baseline; Diff. Abundant ASVs, differentially abundant amplicon sequence variants among the bacterial consortia recovered by fluorescence-activated cell sorting; GDCA, glycodeoxycholic acid; HOMA-IR, homeostatic model assessment of insulin resistance; ILCA, isolithocholic acid; LCA, lithocholic acid; MCC, microcrystalline cellulose; TLCA, taurolithocholic acid; TNF-α, tumor necrosis factor-α

For the effect of AX on satiety after a meal, only RFCs trained on the metabolically active taxa identified by BONCAT resulted in models with significant predictive ability. The best model was obtained with the 14 differentially abundant ASVs (receiver operating characteristic curves [AUC-ROC] = 0.95; Fig. 4A), but the model that was based on the 90 active ASVs still showed a high prediction accuracy (AUC-ROC = 0.84; Fig. 4B). A weak positive correlation that approached significance was detected between propionate-producing *D. invisus *(ASV6pygnt) and satiety (*r*_s_ = 0.63, *q* = 0.08, Spearman’s correlation; Fig. 4A). Satiety was also negatively correlated with formate-producing *Dorea formicigenerans* (ASV2xmw96; *r*_s_ = − 0.81, *q* = 0.007), with butyrate producers *Eubacterium ramulus* (ASV56kx74; *r*_s_ = − 0.60, *q* = 0.08) and *F. prausnitzii* (ASV; *r*_s_ = − 0.56, *q* = 0.098) also showing weak negative correlations (Fig. 4B). *F. prausnitzii* and *D. formicigenerans* further showed increases only in low-responders (*p* < 0.01). Although AX induced fecal propionate [33], which is the SCFA with the strongest evidence for satietogenic effects [22, 37], RFCs based on fecal SCFA shifts could not predict satiety after a meal (OOB error > 0.6) (Additional file 9: Figure S3C).

The only RFC that predicted HOMA-IR was AX-induced shifts in secondary bile acids (AUC-ROC = 0.70; Fig. 4C). MCC-induced fecal calprotectin responses were also predicted by MCC-induced shifts in secondary bile acids (AUC-ROC = 0.72; Fig. 4D), but different secondary bile acids were predictors. Improvements in HOMA-IR showed weak inverse correlations with reductions in the concentrations of lithocholic acid (LCA) (*r*_*s*_ = − 0.62, *q* = 0.08) and its derivative ILCA (*r*_*s*_ = − 0.60, *q* = 0.08) (Fig. 4C), while calprotectin responses were negatively correlated with reductions in conjugated bile acids TLCA (*r*_*s*_ = − 0.68, *q* = 0.03), TDCA (*r*_*s*_ = − 0.70, *q* = 0.03), and GDCA (*r*_*s*_ = − 0.63, *q* = 0.05) (Fig. 4D). A comparison of fiber-induced bile acid shifts revealed significant differences between high-responders and low-responders, with shifts only being detected in low-responders (Fig. 4C and D). For the TNF-α response, only the RFC based on calorie-adjusted intakes of macronutrients at baseline could predict the effect of MCC (AUC-ROC = 0.73; Fig. 4E). While the most discriminative nutrient was saturated fat, consumption at baseline did not associate with TNF-α responses (*r*_*s*_ = 0.46, *q* = 0.17).

To determine whether associations between surrogate endpoints and microbiome features were independent of confounders shown to impact microbiome composition [47], we used multivariate generalized linear models (GLMs) to control for age, sex, changes in total fiber and sugar intake, stool consistency, and bowel movement frequency as covariates (Additional file 10: Table S7). These analyses showed that the observed associations were not confounded by host or dietary factors (*p* < 0.1). As dietary fibers may indirectly influence the intestinal bile acid pool by altering environmental conditions such as pH and water availability [6, 48], links to fecal pH and moisture content were also evaluated (Additional file 11: Figure S4). Though values were not changed by either fiber (*p* > 0.1) [33], positive associations were detected between the shifts in fecal pH (*q* = 0.06) and moisture content (*q* < 0.05) and the bile acid shifts predictive of AX- and MCC-induced effects, respectively.

Our study was specifically designed to evaluate mechanistic associations between fiber-responsive bacterial taxa [33] and mechanistic biomarkers of host-microbiome interactions that relate to fiber fermentation. Although SCFAs, barrier function, and the dominant AX-responsive taxa (*P. copri* and *B. longum*) have been implicated in satiety, insulin resistance, and inflammation [16, 33], significant predictions were not detected (AUC-ROCs < 0.70). Additional univariate GLMs reaffirmed that the dominant fiber-induced shifts in fecal microbiota composition, propionate, and bile acids were not linked to the physiological benefits of fiber supplementation (*q* > 0.05; Additional file 12: Figure S5).

## Discussion

This study established physiological effects for two isolated forms of dietary fiber, AX and MCC, that are therapeutically relevant for individuals with excess weight. By using BONCAT, we provide evidence that the ability of AX to enhance satiety was predicted by bacterial taxa involved in its utilization, suggesting that fermentation of fiber by the gut microbiota may influence its satietogenic effects. In contrast, the AX-induced attenuation of insulin resistance, although linked to fecal bile acid shifts, did not show positive correlations with microbiota features. Interestingly, MCC, which is non-accessible by gut microbiota, showed anti-inflammatory effects in the gut by reducing fecal calprotectin levels that were also predicted by bile acid shifts. Surprisingly, molecular markers of biological processes hypothesized to link metabolic activities of the gut microbiome with host metabolism and immunology (i.e., TMAO, gut hormones, cytokines, and intestinal barrier integrity) were not affected by fermentable AX and could not predict its effects. By exploring the role of the gut microbiota in the effects of physicochemically distinct fibers, our study provides insight as to how fibers may impact satiety and obesity-related surrogate endpoints in humans.

The ability of AX to induce satiety is in agreement with previous research [49] on both long-chain AX supplements [50, 51] and AX-rich whole grains [52–54]. Due to its viscous properties, AX may delay gastric emptying, thereby prolonging postprandial satiety [7]. Our results indicate that microbial fermentation of AX might also contribute to perceived satiety during AX consumption since satiety scores were predicted by the bacterial taxa involved in the breakdown of AX, as detected by BONCAT. We observed significant random forest models (Fig. 4A and B) and weak positive correlations between satiety and propionate producers (*D. invisus* and *B. plebeius*), as well as negative associations with butyrate producers, such as *E. ramulus* and *F. prausnitzii*. The findings are in overall agreement with the analysis of fecal SCFAs, which showed that AX shifts the propionate to butyrate ratio in favor of propionate [33]. Therefore, substrate competition between propionate and butyrate producers may have influenced satiety, as only propionate is shown to be satietogenic in humans [22, 37]. Although additional studies are needed to elucidate exact mechanisms and cross-feeding interactions, our findings provide evidence that the gut microbiota might contribute to the satiety-enhancing properties of AX in humans.

Although BONCAT has the potential to provide a direct assessment of bacterial taxa involved in fiber utilization, the approach is not without limitations. As BONCAT applies an in vitro fermentation, in vivo conditions are unlikely to be accurately replicated, and starting inoculums may have been affected by oxygen exposure during fecal collection, loss of viability during storage, and freeze-thawing. Microbes that do not import amino acids and incorporate them into newly synthesized proteins would also fail to be detected by BONCAT. Being a long-chain highly branched and viscous fiber, the AX is further likely to have behaved differently ex vivo than in the gastrointestinal tract. These limitations may provide an explanation for the failure of *B. longum* and *P. copri* to become metabolically active during the in vitro fermentation (Additional file 8: Table S6), even though these species expanded in vivo through AX [33]. However, the significant random forest models that resulted from including all bacterial ASVs identified by BONCAT support the value of this approach and its application in human nutritional studies. Future research is needed to further explore and refine the application of BONCAT to determine the interactions between dietary compounds and the microbiome, and to improve and validate experimental procedures.

The ability of different types of AX to improve glucose and insulin metabolism is well supported [49, 50, 55, 56] and has resulted in an European Food Safety Authority health claim [10]. The effect size detected in our study was comparable with that in studies that tested alternative strategies aimed to attenuate insulin resistance in individuals with obesity, such as a plant-based diet [40], *Akkermansia muciniphila* [41], or FMT [42]. Compared to these strategies, AX supplements would be more cost-effective and, since it is a food-grade dietary fiber, AX would constitute a promising opportunity for the development of functional foods and beverages. Interestingly, HOMA-IR increased in the MCC group, an effect likely attributable to the elevated sugar consumption during the treatment period. Given that sugar consumption increased equally in both groups (Additional file 3: Table S2), our findings indicate that AX may have counteracted the detrimental effects of sugar on insulin sensitivity.

Anti-inflammatory effects of MCC have, to our knowledge, not yet been reported in humans. These findings are, however, in agreement with research in mice, where very high cellulose diets mitigated chemically induced colitis [57, 58] and improved LPS-induced intestinal permeability [59]. Since the anti-inflammatory effects of MCC are novel in humans, larger studies are needed to confirm our findings and explore whether anti-inflammatory properties are specific to large-particle, highly crystalline cellulose.

Although the physiological effects of AX and MCC differed, both responses were predictable in random forest models through shifts in fecal secondary bile acids, albeit different derivatives. For AX, HOMA-IR responses were inversely associated with shifts in LCA and ILCA, two bile acids that can regulate glucose homeostasis via activating FXR- and TGR5-mediated signaling pathways [24]. Fecal calprotectin responses associated with MCC-induced reductions in TLCA, TDCA, and GDCA. While immunoregulatory properties of these conjugated bile acids remain poorly defined, TLCA may exert anti-inflammatory effects by inhibiting inflammasome signaling [60]. These findings are relevant given the emerging role of bile acids in the regulation of metabolism and immunology [23, 24]. However, despite good predictive power, our correlation analyses identified only negative correlations between physiological benefits and bile acids shifts, and significant shifts in bile acids were only observed in low-responders. Cause-and-effect relationships cannot be unraveled with our correlative data, and it is possible that the negative associations arose through indirect effects of the fibers, such as changes in pH (through AX fermentation) or moisture content (through MCC stimulating fluid secretions) [6, 7, 48], as suggested by our GLMs (Additional file 11: Figure S4). Nevertheless, the lack of positive associations between fiber-induced bile acid shifts and surrogate endpoints suggest that modulation of the bile acid pool does not provide a primary mechanism for the clinical benefits of AX and MCC. The specific effects of the two fibers as they relate to reductions in secondary bile acids are still therapeutically relevant as they provide information on how fibers can selectively manipulate bile acids. Secondary bile acids are considered to be cytotoxic [61]; therefore, the observed reductions might constitute a mechanism by which dietary fibers protect against the development of colon cancer [62].

Through a direct comparison of the effects of microbiota-accessible and non-accessible fibers in a human trial, this study was specifically designed to evaluate the role of the microbiome in the effects of fibers and test commonly hypothesized mechanistic links between microbial fermentation and physiological effects of fibers [16, 17]. For instance, the experimental design allowed us to determine whether fiber-induced shifts in fecal SCFAs were linked to effects on satiety and insulin sensitivity via gut hormones or systemic inflammation through improvements in barrier function [5, 16]. However, despite effects on satiety and insulin resistance through AX, none of the mechanistic biomarkers significantly changed in the AX and MCC groups. Even though this was surprising given that animal models provide convincing evidence for the importance of these mechanisms [4, 5], findings from human studies with fermentable fibers are, at best, inconclusive [63–65]. In addition, even though *B. longum* and *P. copri* were the numerically dominant AX-responders [33] and have been previously linked to improvements in inflammation [66], satiety [36], and insulin sensitivity [20] in humans, these taxa did not correlate with physiological outcomes. A microbiome independent effect is in agreement with the fact that MCC is not accessible to the microbiota, that non-accessible forms of AXs such as psyllium can improve insulin sensitivity [67], and that fibers can improve insulin sensitivity in germ-free mice [32]. Overall, our findings serve as a reminder that certain physiological effects of fiber might be microbiome independent and related to physicochemical attributes of fiber, such as viscosity or fecal bulking [6, 7]. Although we acknowledge the limitations of mechanistic studies in humans [30], as well as the challenges in accurately measuring biomarkers [68, 69] and the small sample size of our study, one has to also consider that some principles of the mechanistic actions of fibers detected in animal models, especially as they relate to the gut microbiota, might not apply to humans.

## Conclusion

In summary, this study provides evidence for the physiological benefits of purified forms of dietary fibers, a notion that has been increasingly questioned in recent nutrition literature [2, 14]. Given the importance of satiety, insulin resistance, and systemic inflammation in the etiology of obesity and cardiometabolic diseases, our findings establish the potential role of purified fibers in the prevention and treatment of chronic diseases, while also warranting future studies to explore the anti-inflammatory effects of MCC. The distinct effects of the two fiber types can serve as a basis for a more targeted application of dietary fiber, such as AX for type II diabetes and MCC for inflammatory diseases, which could ultimately inform nutritional guidelines. Our findings also provide evidence for the role of fiber-microbiome interactions in inducing satiety, while the metabolic and immunological effects of the fibers may be primarily microbiota-independent. A better understanding of mechanisms by which fibers induce physiological effects in humans would contribute to a conceptual framework for the development of fiber structures or designer carbohydrates with improved clinical efficacy. In this respect, our findings that the effects of AX on satiety were linked to the ability of an individual’s microbiome to utilize the fiber provides a mechanistic basis to optimize fiber applications through a personalized approach.

## Methods

### Study design

#### Registration

As previously described [33], this randomized controlled exploratory trial was prospectively registered in July 2015 at ClinicalTrials.gov (NCT02322112) as part of a large clinical trial aimed at comparing the effects of four structurally distinct dietary fibers on the gut microbiota and human health. In response to requests by reviewers of a grant application, which advised against including a premarket ingredient in a large human trial, the AX arm was separated from the original trial in October 2016. Data from the participants that completed the AX protocol were analyzed independently and compared to data from the participants that completed the MCC protocol (microbiota-non-accessible controls) during the same period.

#### Dietary intervention

In brief, 38 individuals with excess weight (BMI: 25–35 kg/m^2^) were enrolled and randomly assigned to supplement their habitual diet with either AX or MCC at a daily dose of 25 g (females) or 35 g (males), an amount that resembles Health Canada recommendations for the intake of dietary fiber [70] (Fig. 1). Of the 19 individuals enrolled per arm, 15 participants completed the AX protocol, and 16 participants completed the MCC protocol [33]. The AX used was AGRIFIBER SFC (previously named BIOFIBER GUM), a fermentable, long-chain AX isolated from corn bran (AgriFiber Solutions LLC, USA), while the MCC was MICROCEL MC-12, a non-accessible, large-particle (160-micron average) wood-derived cellulose (Blanver Farmoquimica LTDA, Brazil) [33]. The rationale to focus on a BMI range of 25–35 kg/m^2^ was that these individuals would be more likely to have elevated levels of risk factors that predict cardiometabolic disease, but less likely to have these chronic diseases [21, 71]. Our overall goal was to explore if dietary supplements could attenuate these risk factors prior to disease onset.

### Dietary intake

To assess whether dietary fiber supplementation influenced dietary intake, participants completed two non-consecutive 24-h dietary recalls at baseline and weeks 3 and 6 (Fig. 1) using the Canadian version of the web-based Automated Self-Administered 24-hour Dietary Assessment Tool [72]. Mean values of baseline (two recalls) and of weeks 3 and 6 (four recalls) were used in statistical analyses. Prior to assessing associations, diet data were first calorie-adjusted as previously described [73].

### Perceived satiety

Perceived satiety was evaluated at baseline and weekly during the intervention using the validated SLIM questionnaire (Fig. 1), which is a 100 mm, bidirectional hunger-fullness scale anchored by “greatest imaginable fullness” (50 mm) and “greatest imaginable hunger” (− 50 mm), with “neither hungry nor full” in the center (0 mm) [74]. Each week, a SLIM scale was completed within (*i*) 30 mins of waking and (*ii*) 30–60 min of consuming a meal with AX or MCC added. For between-group comparisons, the area under the SLIM curve (AUC_BL–W6_) was calculated by using the linear trapezoidal method in GraphPad Prism. When applicable, missing data points were imputed using the mean of the participants’ known values, as previously described [75].

### Obesity-related surrogate endpoints

Blood pressure was measured at baseline and week 6 with an automatic sphygmomanometer (Welch Allyn, Hill-Rom Inc., Indiana, USA). Blood samples were also collected at baseline and week 6 after a 12-h overnight fast using separation tubes, K_2_EDTA-coated tubes, and P800 tubes to obtain serum, plasma, and inhibitor-treated plasma, respectively. P800 tubes are pre-coated with K_2_EDTA plus a proprietary cocktail of protease, esterase, and DPP-IV inhibitors to prevent hormone degradation (BD Biosciences, USA). Total cholesterol, high-density lipoprotein cholesterol, triglycerides, and glucose were then quantified in serum on a Beckman Coulter DxC 800 (Beckman Coulter Inc., California, USA), with insulin measured in inhibitor-treated plasma by electrochemiluminescence immunoassay (ECLIA; K15174C, MesoScale Discovery^®^, Maryland, USA; intra-assay coefficient of variation [CV] 4.5%). Low-density lipoprotein cholesterol, HOMA-IR, and QUICKI values were then calculated as previously described [76–78]. To assess intestinal and systematic inflammation, fecal calprotectin and plasma high-sensitivity C-reactive protein were quantified by enzyme-linked immunosorbent assay (ELISA; K6927, Immundiagnostik AG, Bensheim, Germany) and ECLIA (K151STD; CV 2.1%), respectively. Finally, whole blood was collected for immediate quantification of complete blood count parameters using a Sysmex XN-10 analyzer (Sysmex Corporation, Kobe, Japan).

### Biomarkers of host-microbiome interactions implicated in the pathophysiology of obesity

#### Appetite, glucose metabolism, and systemic inflammation

Several hormonal regulators of appetite and glucose metabolism were quantified in inhibitor-treated plasma by ECLIA (MesoScale Discovery^®^). This included total ghrelin and peptide YY by a U-PLEX^®^ Assay (K151ACL; CV 3.2% and 2.4%), along with active glucagon-like peptide-1, glucagon, and leptin by a MULTI-SPOT^®^ Assay (K15174C; CV 6.6%, 6.0%, and 5.1%). In addition, both adipocyte-derived hormone adiponectin, and the cytokines TNF-α, interleukin*-*6, interleukin-8, and interleukin-10 were measured in plasma by single-plex (K151BXC; CV 3.3%) and multiplex (K15049D; CV 3.0%, 4.3%, 3.7%, and 5.8%) assays, respectively.

#### Intestinal barrier integrity

Lipopolysaccharide-binding protein, albumin, and zonulin were quantified by ELISA as measures of gut barrier function recently validated by our group [79]. While lipopolysaccharide-binding protein was measured in plasma diluted 1:1300 in phosphate-buffered saline (PBS) (SEB406Hu, USCN Life Science and Technology, Texas, USA; CV 12.6%), albumin and zonulin were measured in fecal samples prepared by the Stool Sample Application System (K6330, K5600, and K6998SAS, Immundiagnostik AG), which diluted samples 1:100 in a proprietary extraction buffer.

#### TMAO

TMAO, a metabolite generated in the liver from the oxidation of bacterial-derived trimethylamine (TMA), was measured in serum by high-performance liquid chromatography-tandem mass spectrometry as previously described [80], with a CV of 5.0%. Briefly, for TMAO extraction, 50 μL of thawed serum was spiked with 50 μL of internal standard solution (TMAO-d9 and TMA-d9) and 150 μL of methanol with 0.1% formic acid. The mixture was then vortexed for 1 min and centrifuged at 10,000 rpm for 15 min at 15 °C. The supernatant was collected and stored frozen at − 20 °C until analysis. For sample derivatization, 25 μL of supernatant was reacted with 50 μL of ethyl bromoacetate (4 mg/ml in acetonitrile) in the presence of 3 μL concentrated ammonium hydroxide for 40 min at room temperature. Then, HPLC-grade water containing 0.5% formic acid was added to obtain the final volume of 500 μL and then stored at – 20 °C until analysis.

An Agilent 1200 series HPLC system (Agilent Technologies Inc., California, USA) coupled to a 3200 QTRAP mass spectrometer (AB SCIEX, Ontario, Canada) was used under turbospray positive mode to analyze standard and sample solutions. An Ascentis Express HILIC column (15 cm × 2.1 mm, 2.7 μm particle size; Sigma, Missouri, USA) was used at room temperature for LC separation. Composition of the mobile phase used for isocratic elution was (solvent A) 0.1% formic acid in acetonitrile and (solvent B) 10 mM ammonium formate (70:30, v/v). The run time was set as 6 min with a flow rate of 0.25 mL/min. Electrospray ionization was used under positive-ion detection with multiple-reaction monitoring scans. All other instrumental parameters used were as follows: curtain gas at 20 arbitrary units; gas 1 at 50; gas 2 at 60; ion spray voltage at 5200 V. The dwell time for each transition ion was 300 ms and the ion source temperature was 400 °C. Multiple-reaction monitoring transitions used were as follows: TMA derivative: 146.1 > 118.1; TMAO: 76.1 > 58.1; TMA-d9 derivative: 155.1 > 127.1; TMAO-d9: 85.1 > 68.1.

#### Fecal bile acids

Fecal bile acids were characterized by targeted metabolomics of 60 bile acids with ultrahigh performance liquid chromatography/multiple-reaction monitoring-mass spectrometry at the University of Victoria Genome British Columbia Proteomics Centre as previously described [81]. Briefly, bile acids were extracted from lyophilized and homogenized fecal samples by adding 1 mL of 75% acetonitrile to 10 mg of sample, followed by 20 sec of vortexing at 3,000 rpm, 5 min of sonication in an ice water bath, and 5 sec of additional vortexing. Samples were centrifuged at 15,000 rpm and 10 °C for 15 min, and then 20 μL of the supernatant was mixed with 60 μL of 50% methanol and 40 μL of internal standard. Finally, 10 μL of the mixture was injected onto an Agilent 1290 series HPLC system coupled to a 4000 QTRAP mass spectrometer. A Waters BEH C18 UPLC column (Waters Corp., Massachusetts, USA) was used for chromatographic separation. Composition of the mobile phase used for binary-solvent gradient elution was 0.01% formic acid in water (solvent A) and 0.01% formic acid in acetonitrile (solvent B). The flow rate was 0.35 mL/min with the column temperature maintained at 45 °C.

### Ex vivo characterization of the bacterial consortia that utilize AX

#### Collection and processing of fecal samples

Fecal samples were collected at 6 weeks of AX supplementation as previously described [33]. Briefly, fecal material was collected in a stool specimen container and placed in an air-tight bag that contained a GasPak™ EZ Anaerobe Sachet (BD, Canada) to maintain anaerobicity. Samples were brought to the clinic within 4 h of defecation and processed immediately in a Bactron Anaerobic Chamber (Shel Lab, Oregon, USA) under anaerobic conditions (5% H_2_, 5% CO_2_, and 90% N_2_). Fecal material used for BONCAT was then diluted 1:10 in pre-reduced molecular grade PBS with 10% glycerol (a method previously shown to maintain microbiota viability [82]), aliquoted, and stored at − 80 °C until being shipped frozen on dry ice to the University of Vienna for further analysis.

#### BONCAT of AX-utilizing bacterial cells

To identify bacterial taxa involved in the degradation of AX and utilization of breakdown products released during degradation, we applied BONCAT [39], a fluorescence-based single-cell labeling of cellular activity, to week 6 fecal samples from the AX treatment group (Fig. 3). Briefly, BONCAT is based on incorporating the non-canonical amino acid AHA instead of *L*-methionine during protein synthesis, followed by fluorescent labelling of AHA-containing cellular proteins by azide-alkyne click chemistry. Using a Cu(I)-catalyzed reaction, a terminal alkyne coupled fluorescence dye, such as Cy5, can be linked to the azide group of the incorporated AHA; thus, marking microbial cells that have undergone protein synthesis during incubation with AHA and AX [83].

To apply BONCAT, fecal homogenates were first thawed and introduced into an anaerobic tent (85% N_2_, 10% CO_2_, 5% H_2_) upon arrival to the University of Vienna, with all reagents and laboratory consumables being introduced into the anaerobic tent two days prior to the experiment to ensure anaerobicity at the start of the experiment. Fecal homogenates were filtered (40 μm filter, Corning, Germany) to remove particles, washed twice in 1× PBS to remove residual glycerol, and diluted 1:10 in 1× PBS, as opposed to nutrient-rich media, to limit background noise, avoid autofluorescence in the Cy5 dye solution, and select for bacterial cells that preferentially utilize AX. Samples were then added to sterile Hungate tubes with 1 mM of cellular activity marker AHA (Baseclick GmbH, Germany) and 2 mg/mL of in vitro pre-digestion AX [84] (consistent with dietary intakes of AX [85, 86]), and then incubated in an anaerobic tent at 37 °C for 6 h. For each sample, a non-amended negative control, wherein only 1 mM of AHA was added, was also incubated to account for potential basal activity, of which there was no basal activity detected. After 6 h of incubation, biomasses were washed with 1× PBS, fixed in ethanol, and stored at – 20 °C in 1:1 ethanol/PBS.

To prepare the Cy5 dye solution, 1.25 μl of 20 mM CuSO_4_, 2.50 μl of 50 mM THPTA (Baseclick, Germany), and 0.30 μl of Cy5 alkyne dye (Jena Bioscience, Germany) were left in the dark for 3 min to react and then added to 221 μl of 1× PBS, 12.5 μl of 100 mM sodium ascorbate (Sigma-Aldrich, Austria), and 12.5 μl of 100mM aminoguanidine hydrochloride (Sigma-Aldrich, Austria). Next, 300–500 μl of the fixed biomasses were centrifuged at 10,000 rpm for 10 min and resuspended in 96% ethanol once the supernatants were removed. Finally, 60–100 μl of the dye solution was added to the fixed biomasses, incubated in the dark at room temperature for 30 min, washed three times with 1× PBS, and then filtered with 35 μm nylon mesh using 12 × 75 mm BD tubes (BD, Germany) immediately before being sorted by flow cytometry. Biomasses were also collected from the amended samples at 0- and 6-h incubations for additional DNA extractions.

#### FACS of AX-utilizing bacterial cells

Flow cytometry FACS of Cy5-labeled bacterial cells was done with an ultra-high-speed cell sorter MoFlo Astrios EQ (Beckman Coulter, California, USA) using the software Summit v6.2 (Beckman Coulter), as represented in Additional file 7: Figure S2. To standardize measurements and assess bacterial size, silica calibration beads (100, 500, and 1000 nm, Kisker Biotech, Germany) with refractive indexes close to that of biological material were recorded. The sorting of Cy5-labeled bacteria was performed as followed: background noise of the machine was first detected using the parameters forward scatter and side scatter. 488-nm FSC1-Height-Log vs 488-nm SSC2-Height-Log was then used to show the different sizes of silica beads in the first measurement and the scattering of the bacteria in subsequent measurements. Bacteria were pre-gated and displayed on a third scatter plot with 488 nm SSC-Area-Log vs 640 nm 671/30-Area-Log axes. Cy5-positive bacteria were then sorted out into tubes with a maximum event rate of 50,000 events/s. Reanalysis of the samples showed a purity of more than 99%.

#### DNA extraction, 16S rRNA gene amplicon sequencing, and inference of bacterial ASVs

Bacterial DNA from both FACS-sorted cells (6 h) and fecal incubations (0 and 6 h) were extracted using QIAamp DNA Mini Kit (Qiagen, Germany) following the protocol for bacteria according to the manufacturer’s instructions. Cell lysis was further performed enzymatically by first using Proteinase K and then by a second lysozyme step (Sigma-Aldrich, Austria). The V3–V4 region of the 16S rRNA gene was amplified and barcoded using a previously described 2-step PCR approach [87] with 16S rRNA gene primers S-D-Bact-0341-b-S-17 (5′-CCTACGGGNGGCWGCAG-3′) and S-D-Bact-0785-a-A-21 (5′-GACTACHVGGGTATCTAATCC-3′) [88]. Barcoded samples were then purified and normalized over a SequalPrep™ Normalization Plate Kit (Invitrogen) using a Biomek^®^ NXP Span-8 pipetting robot (Beckman Coulter, California, USA), then pooled and concentrated on columns (Anlaytik Jena). Next, sequence libraries were prepared with the Illumina TruSeq Nano Kit as previously described [87] by sequencing in paired-end mode (2 × 300 nt; v3 chemistry) on an Illumina MiSeq. After sequencing, amplicon pools were extracted from the raw sequencing data using the FASTQ workflow in BaseSpace (Illumina) with default parameters, and then sequences were demultiplexed with the python package demultiplex [89] by permitting one mismatch each for barcodes, linkers, and primers. Contaminants, including mitochondria and chloroplast sequences, were removed using the R package decontam v1.6.0 [90] with the prevalence method and a threshold setting of 0.01. Sequence data were then analyzed using a bioinformatic approach based on ASVs as described previously [39, 91].

### Statistical analyses

#### Statistical assessment of clinical effects

Statistical analyses were performed using R v3.5.1, Stata v15.0, and GraphPad Prism v9.1.2. Prior to statistical analyses, outliers were identified and removed based on a mean ± five standard deviation cutoff (≤ 2 participants per endpoint) [92]. To assess the overall effects of fiber supplementation on perceived satiety and surrogate endpoints, data were ordinated by principal component analysis using factoextra [93] and FactoMineR [94] packages. Then, between-group differences (AX-vs-MCC) were assessed by permutational multivariate analysis of variance based on Manhattan distances [95] using the Adonis function in the vegan [96] package. For perceived satiety, surrogate endpoints, biomarkers (apart from SCFAs [33]), and diet variables, repeated measures one-way ANOVA and paired *t-*tests with permutations (*n *= 1000) were applied to compare within-group differences relative to baseline using the permuco [97] package. Between-group differences were assessed by unpaired permutational *t*-tests (*n *= 1000) using the lmPerm [98] package. For BONCAT identified bacterial ASVs, the DEseq2 [99] package was used to identify ASVs whose abundance differed in the bacterial consortia recovered by FACS after a 6-h incubation in the presence of AX as compared to the total fecal bacterial communities after 0- and 6-h incubations. For surrogate endpoints, mechanistic biomarkers, and bacterial ASVs, a more stringent cutoff of *p* < 0.01 was considered significant to account for multiple comparisons and to detect only the robust effects of AX and MCC consumption, while *p* < 0.05 was considered significant for the remaining analyses where relatively few comparisons were made.

#### Machine learning models to predict clinical effects

To identify potential determinants of host-microbiome interactions that predicted the effects of fiber supplementation on perceived satiety and surrogate endpoints, separate RFCs were independently trained on changes in bacterial composition, ecological variables of the broader bacterial community (as previously determined [33]), mechanistic biomarkers, and macronutrient intake datasets (refer to Additional file 13: Table S8 for a description of each predictor dataset). Random forest uses supervised tree-based machine learning algorithms that are purported to be a robust approach for the discriminant analysis of high dimensional, low sample size data [100–102]. Prior to analysis, participants were classified as high- or low-responders for each endpoint according to the study cohort median, as in satiety after a meal (AUC_BL–W6_), HOMA-IR, fecal calprotectin, and TNF-α (percentage change). Since HOMA-IR and QUICKI indexes showed significant collinearity (*r*_s_ = − 0.97 and *p* < 0.0001, Pearson’s correlation) with the same classifications, RFC analyses were only performed on HOMA-IR.

Independent RFCs were performed using the default settings in the randomForest [103] package, with the generalization error of each RFC estimated across 100 replicates using the leave-one-out cross-validation as previously described [104]. To evaluate the performance of each RFC, AUC-ROCs were generated from the true possible cross-validated results using the pROC [105] package and average out-of-bag error rates were estimated across 100 replicates. RFCs with AUC-ROC values ≥ 0.7 and out-of-bag error rates < 0.6 were considered to have good prediction accuracy [102]. A confusion matrix was further generated to evaluate subgroup prediction accuracy. To determine the importance of each individual variable for the classification of high-vs-low-responders, average mean importance scores were calculated by 100 replicates estimation.

To support the RFCs, Spearman’s correlations were performed between the endpoint and its best predictors, which showed the directionality of the associations. Univariate GLMs were further performed between the endpoint and the dominant fiber-induced shifts in fecal microbiota composition, propionate, and bile acids observed. To account for multiple comparisons, false discovery rate adjusted *q* values < 0.05 were considered significant. Data distributions were visually assessed by inspection of residual and histogram plots. Non-normally distributed data were cubed-root transformed prior to analysis by Gaussian-distribution GLMs with the identity link. Binominal-distribution GLMs with the logistic link were alternatively applied for HOMA-IR, as percentage change data were binomially distributed.

#### Adjusting for potential confounding effects

ANCOVA models were used to adjust for covariates that may have confounded the observed clinical effects. Multivariate GLMs were alternatively used to adjust for covariates that may have confounded the associations detected between surrogate endpoints and microbiome markers. Due to limitations in statistical power (small sample size), separate ANCOVA models and GLMs were performed for each covariate. Statistical significance was considered at *p* < 0.05 as relatively few comparisons were made.

## Supplementary Information

**Additional file 1: Figure S1.** Adherence to the study protocol as estimated by the average amount (weight) of dietary fiber consumed during the intervention period.

**Additional file 2: Table S1.** Baseline clinical measurements. Table provides the anthropometric measurements, surrogate endpoints, and biomarkers of host-microbiota interactions assessed at baseline, with participant grouped by arabinoxylan or microcrystalline cellulose supplementation. Data provided as mean ± SD.

**Additional file 3: Table S2.** Macronutrient consumption at baseline and during arabinoxylan or microcrystalline cellulose supplementation. Table provides total dietary calories and macronutrients as assessed by the Canadian version of the web-based Automated Self-Administered 24-hour Dietary Assessment Tool, with participant grouped by arabinoxylan or microcrystalline cellulose supplementation. Data provided as mean ± SD.

**Additional file 4: Table S3.** Clinical measurements at baseline and six weeks of arabinoxylan or microcrystalline cellulose supplementation. Table provides the anthropometric measurements, surrogate endpoints, biomarkers of host-microbiota interactions, and stool characteristics assessed, with participant grouped by arabinoxylan or microcrystalline cellulose supplementation. Data provided as mean ± SD.

**Additional file 5: Table S4.** Covariate-adjustment of dietary fiber treatment effects by analysis of covariance (ANCOVA). Table provides *p* values from separate ANCOVA models used to adjust for the following covariates: age, sex, total dietary fiber intake, total dietary sugar intake, stool consistency, and bowel movement frequency.

**Additional file 6: Table S5.** Fecal concentrations of remaining bile acids detected at baseline and six weeks of arabinoxylan or microcrystalline cellulose supplementation. Table provides the remaining 29 bile acid compounds that were detected in < 90% of fecal samples, with participant grouped by arabinoxylan or microcrystalline cellulose supplementation.

**Additional file 7: Figure S2.** Sorting of AX-stimulated bacterial cells by FACS on a MoFlow Astrios EQ cell sorter. As shown in the dot plots, **A** background noise of the machine was detected using FSC and SSC parameters. **B** Bacterial cells were measured in the same setting and pre-gated. **C** An example of Cy5-negative cells is presented in the dot plot showing the Cy5 channel via the SSC channel. **D** An example of Cy5-positive fluorescent cells (activated by AX) that were gated and sorted out by FACS. AX, arabinoxylan; FACS, fluorescence-activated cell sorting; FSC, forward scatter; SSC, side scatter.

**Additional file 8: Table S6.** Relative abundance of bacterial taxa activated through ex vivo incubation with arabinoxylan and recovered by fluorescence-activated cell sorting (FACS). Table provides α-diversity and the bacterial taxa that were activated at 6-h incubation with arabinoxylan, recovered by FACS, showed a mean relative abundance > 1%, and/or showed a differential abundance in the recovered consortia relative to the total fecal communities. Data provided as mean ± SD.

**Additional file 9: Figure S3.** Confirmation of gut microbiota compositional features and mechanistic biomarkers that predict clinical responses. Line graphs show differences in the effects of **A** AX on perceived satiety after a meal and HOMA-IR and **B** MCC on fecal calprotectin and TNF-α for high and low responders, as defined according to the study cohort median. AUC-ROC values show the performance accuracy of random forest classifiers for predicting high-vs-low responders in **C** AX-induced perceived satiety after a meal and HOMA-IR attenuation, **D** MCC-induced fecal calprotectin and TNF-α attenuation, and **E** AX and MCC induced changes in HOMA-IR, fecal calprotectin, and TNF-α. High and low responders were defined according to the study cohort median. Black cells denote OOB error rates ≥ 0.6. Prediction performance of random forest classifiers trained to predict high-vs-low responders in AX-induced **F** and **G** satiety after a meal and **H** HOMA-IR attenuation, and MCC-induced **I** fecal calprotectin and **J** TNF-α attenuation. OOB shows the mean prediction error of the random forests model with boosted decision trees (*n *= 500). The confusion matrix shows subgroup prediction accuracy, where row *i* and column *j* indicates the number of subjects predicted as *i* but were actually classified as *j*. Error rates indicate the percentage of incorrect classifications. ∆, absolute change from baseline to week 6; %∆, percent change from baseline to week 6; ASV, amplicon sequence variant; AX, arabinoxylan; AUC-ROC, area under the receiver operating characteristic curve; HOMA-IR, homeostatic model assessment of insulin resistance; MCC, microcrystalline cellulose; OTU, operational taxonomic unit; OOB: out-of-bag; SLIM, Satiety Labeled Intensity Magnitude; TNF-α, tumor necrosis factor-α.

**Additional file 10: Table S7.** Univariate and covariate-adjusted generalized linear models (GLMs) assessing fecal microbiota-related factors that associate with surrogate endpoints of dietary fiber supplementation. Table provides β-coefficient directionality and *p* values from separate GLMs used to adjust for the following covariates: age, sex, total dietary fiber intake, total dietary sugar intake, stool consistency, and bowel movement frequency.

**Additional file 11: Figure S4.** Scatter plots show Spearman’s correlations between shifts in **A **and **B **fecal pH and **C **and **D **fecal moisture content and changes in the fecal concentrations of bile acids shown to predict the arabinoxylan and microcrystalline cellulose induced reductions in HOMA-IR and fecal calprotectin, respectively. Statistical significance was set at FDR adjusted *q* values < 0.05. GDCA, glycodeoxycholic acid; HOMA-IR, homeostatic model assessment of insulin resistance; ILCA, isolithocholic acid; LCA, lithocholic acid; TDCA, taurodeoxycholic acid; TLCA, taurolithocholic acid.

**Additional file 12: Figure S5.** Associations between the effects on perceived satiety and surrogate endpoints and the dominant fecal microbiota features affected by fiber supplementation. Heatmap shows cubed-root transformed β-coefficients of univariate generalized linear models performed on the compositional (dominant AX-responsive taxa at baseline, shifts, and ex vivo) and functional (fecal propionate and bile acid shifts) features of the gut microbiota. Statistical significance was considered at FDR corrected *q* values < 0.05. ∆, absolute change from baseline to week 6; %∆, percent change from baseline to week 6; 7αOHCA; 7αOH-3-oxo-4-cholestenoic acid; ApoCA; apocholic acid; ASV, amplicon sequence variant; AUC, area under the curve; AX, arabinoxylan; BL, baseline; HDCA, hyodeoxycholic acid; HOMA-IR, homeostatic model assessment of insulin resistance; MCC, microcrystalline cellulose; OTU, operational taxonomic unit; TNF-α, tumor necrosis factor-α.

**Additional file 13: Table S8.** Description of predictor datasets used for training the independent random forest classifiers. Table provides both a brief and detailed description of the variables included in each predictor dataset used for the random forest analyses, as referenced in Fig. 4 and Additional file 9: Figure S3.

## Data Availability

The 16S rRNA gene amplicon sequencing data were deposited in the NCBI Sequence Read Archive and are available for download under BioProjects PRJNA564636 (fecal) and PRJNA630848 (ex vivo). Remaining deidentified individual participant data, methods, and study materials will be made available from the corresponding authors upon reasonable request.

## Abbreviations

ANCOVA: Analysis of covariance
ASV: Amplicon sequence variant
AUC-ROC: Area under the receiver operating characteristic curve
AX: Arabinoxylan
BMI: Body mass index
BONCAT: Bioorthogonal non-canonical amino acid tagging
CV: Intra-assay coefficient of variation
ECLIA: Electrochemiluminescence immunoassay
ELISA: Enzyme-linked immunosorbent assay
FACS: Fluorescence-activated cell sorting
FMT: Fecal microbiota transplantation
GDCA: Glycodeoxycholic acid
GLM: Generalized linear model
HOMA-IR: Homeostatic model assessment of insulin resistance
ILCA: Isolithocholic acid
LCA: Lithocholic acid
MCC: Microcrystalline cellulose
PBS: Phosphate-buffered saline
QUICKI: Quantitative insulin sensitivity check index
RFC: Random forest classifier
SCFA: Short-chain fatty acid
SLIM: Satiety Labeled Intensity Magnitude
TDCA: Taurodeoxycholic acid
TLCA: Taurolithocholic acid
TMA: Trimethylamine
TMAO: Trimethylamine *N*-oxide
TNF-α: Tumor necrosis factor-α
