# Supplementary figures and images for: Elucidating the role of the gut microbiota in the physiological effects of dietary fiber
Source: Microbiome. 2022 May 13;10:77. doi: 10.1186/s40168-022-01248-5 (PMC9107176; doi:10.1186/s40168-022-01248-5)

**Additional file 1: Figure S1.**

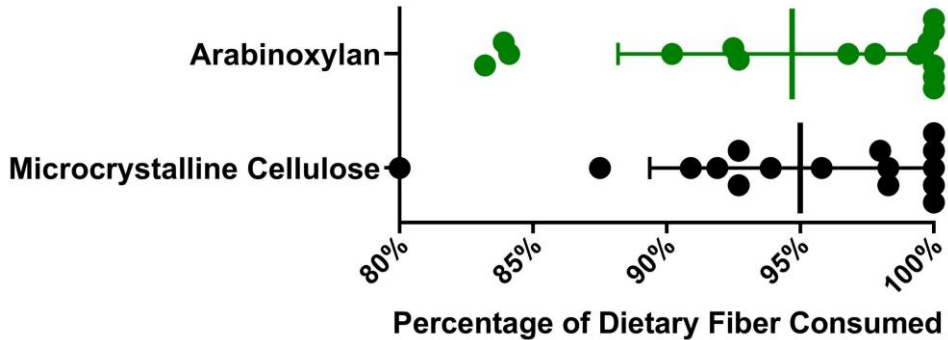

Supplement: Supplementary file 2 — Additional file 1: Figure S1. Adherence to the study protocol as estimated by the average amount (weight) of dietary fiber consumed during the intervention period. [file 40168_2022_1248_MOESM1_ESM.pdf]

**A** Background Noise

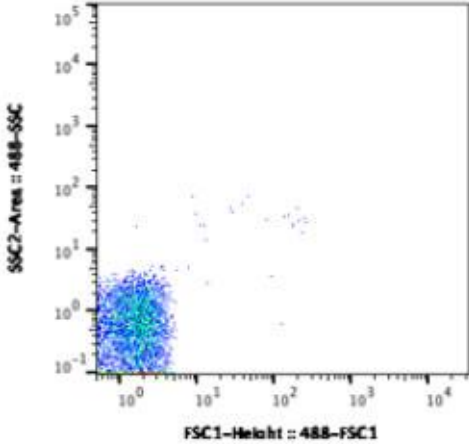

**B** Scatter

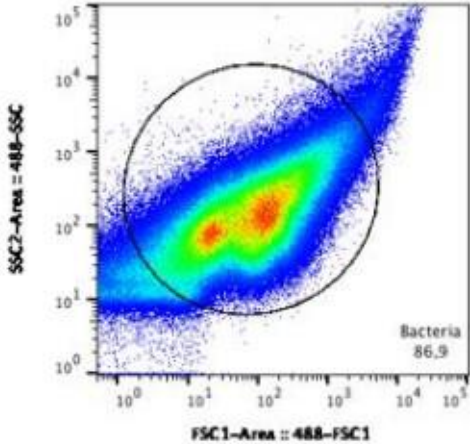

**C** Cy5-Negative

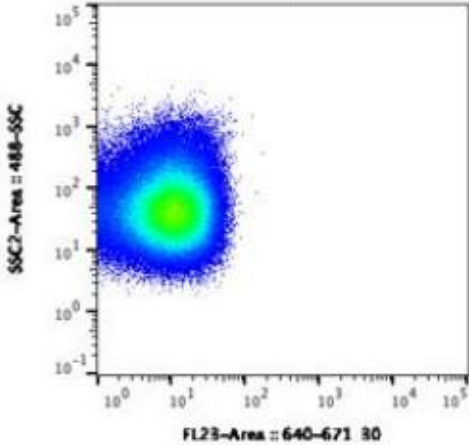

**D** Cy5-Positive

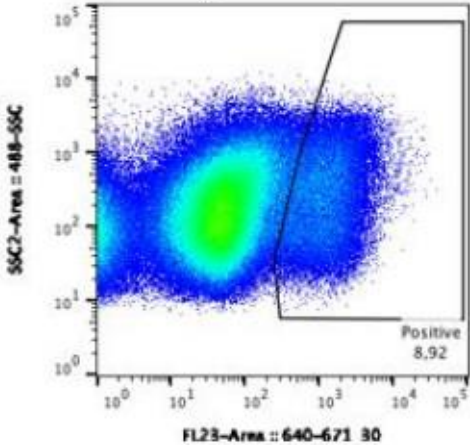

Supplement: Supplementary file 8 — Additional file 7: Figure S2. Sorting of AX-stimulated bacterial cells by FACS on a MoFlow Astrios EQ cell sorter. As shown in the dot plots, A background noise of the machine was detected using FSC and SSC parameters. B Bacterial cells were measured in the same setting and pre-gated. C An example of Cy5-negative cells is presented in the dot plot showing the Cy5 channel via the SSC channel. D An example of Cy5-positive fluorescent cells (activated by AX) that were gated and sorted out by FACS. AX, arabinoxylan; FACS, fluorescence-activated cell sorting; FSC, forward scatter; SSC, side scatter. [file 40168_2022_1248_MOESM7_ESM.pdf]

# Additional file 9: Figure S3.

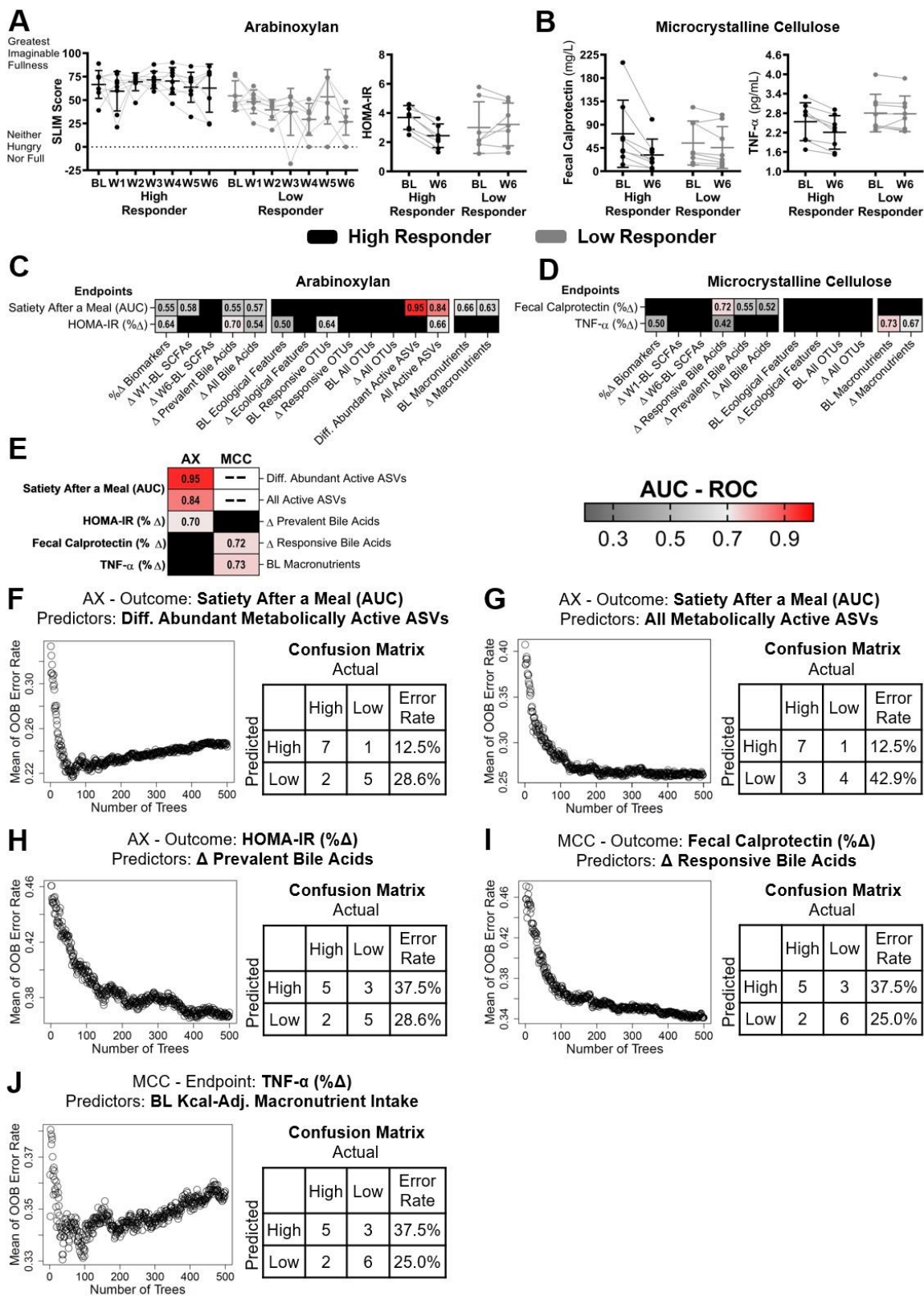

Supplement: Supplementary file 10 — Additional file 9: Figure S3. Confirmation of gut microbiota compositional features and mechanistic biomarkers that predict clinical responses. Line graphs show differences in the effects of A AX on perceived satiety after a meal and HOMA-IR and B MCC on fecal calprotectin and TNF-α for high and low responders, as defined according to the study cohort median. AUC-ROC values show the performance accuracy of random forest classifiers for predicting high-vs-low responders in C AX-induced perceived satiety after a meal and HOMA-IR attenuation, D MCC-induced fecal calprotectin and TNF-α attenuation, and E AX and MCC induced changes in HOMA-IR, fecal calprotectin, and TNF-α. High and low responders were defined according to the study cohort median. Black cells denote OOB error rates ≥ 0.6. Prediction performance of random forest classifiers trained to predict high-vs-low responders in AX-induced F and G satiety after a meal and H HOMA-IR attenuation, and MCC-induced I fecal calprotectin and J TNF-α attenuation. OOB shows the mean prediction error of the random forests model with boosted decision trees (n = 500). The confusion matrix shows subgroup prediction accuracy, where row i and column j indicates the number of subjects predicted as i but were actually classified as j. Error rates indicate the percentage of incorrect classifications. ∆, absolute change from baseline to week 6; %∆, percent change from baseline to week 6; ASV, amplicon sequence variant; AX, arabinoxylan; AUC-ROC, area under the receiver operating characteristic curve; HOMA-IR, homeostatic model assessment of insulin resistance; MCC, microcrystalline cellulose; OTU, operational taxonomic unit; OOB: out-of-bag; SLIM, Satiety Labeled Intensity Magnitude; TNF-α, tumor necrosis factor-α. [file 40168_2022_1248_MOESM9_ESM.pdf]

Additional file 11: Figure S4.

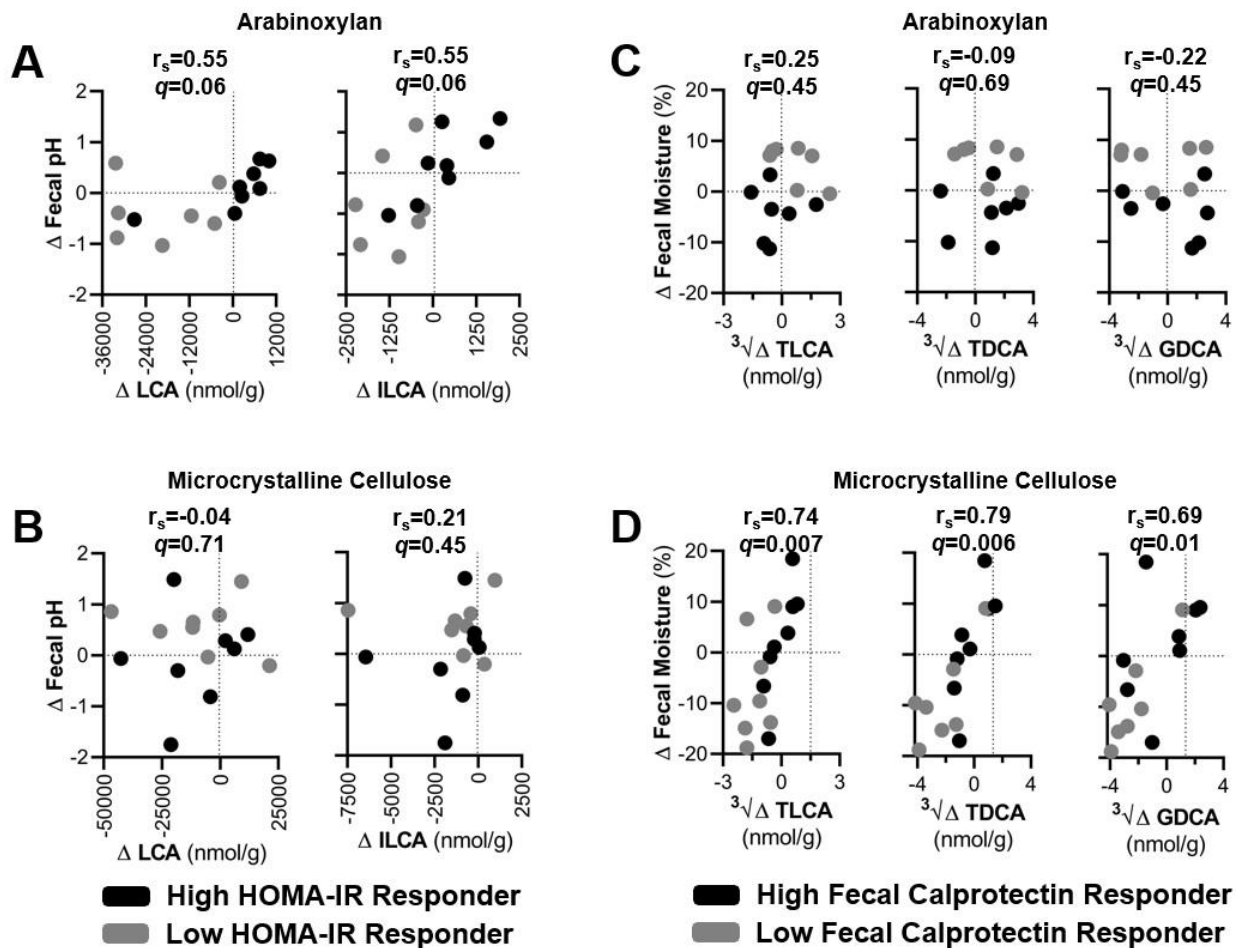

Supplement: Supplementary file 12 — Additional file 11: Figure S4. Scatter plots show Spearman’s correlations between shifts in A and B fecal pH and C and D fecal moisture content and changes in the fecal concentrations of bile acids shown to predict the arabinoxylan and microcrystalline cellulose induced reductions in HOMA-IR and fecal calprotectin, respectively. Statistical significance was set at FDR adjusted q values < 0.05. GDCA, glycodeoxycholic acid; HOMA-IR, homeostatic model assessment of insulin resistance; ILCA, isolithocholic acid; LCA, lithocholic acid; TDCA, taurodeoxycholic acid; TLCA, taurolithocholic acid. [file 40168_2022_1248_MOESM11_ESM.pdf]

**Additional file 12: Figure S5.**

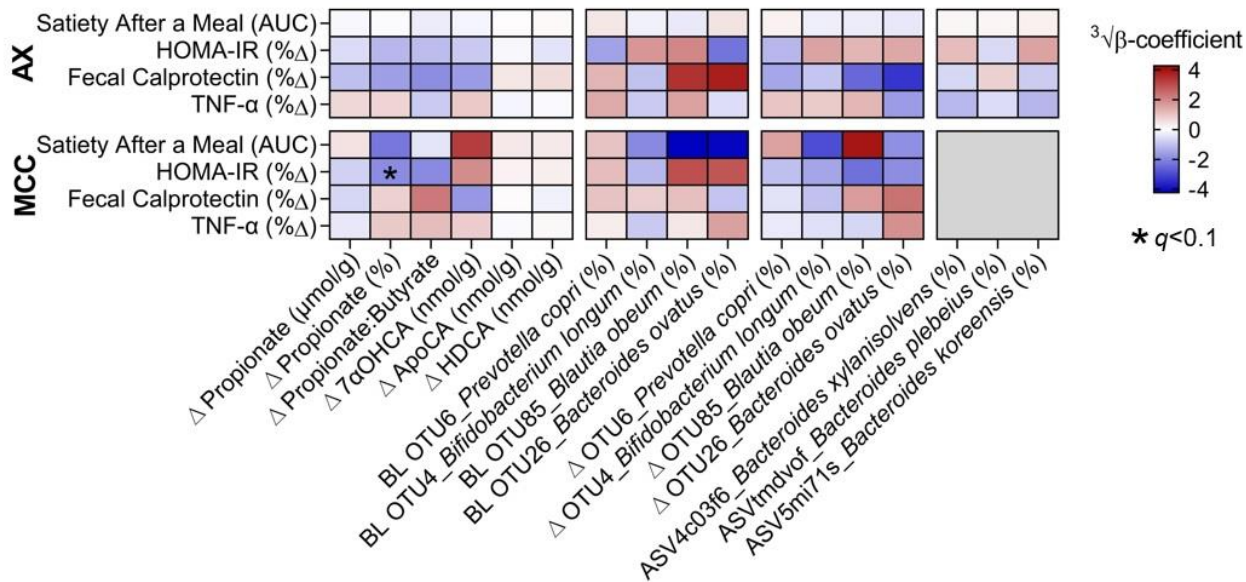

Supplement: Supplementary file 13 — Additional file 12: Figure S5. Associations between the effects on perceived satiety and surrogate endpoints and the dominant fecal microbiota features affected by fiber supplementation. Heatmap shows cubed-root transformed β-coefficients of univariate generalized linear models performed on the compositional (dominant AX-responsive taxa at baseline, shifts, and ex vivo) and functional (fecal propionate and bile acid shifts) features of the gut microbiota. Statistical significance was considered at FDR corrected q values < 0.05. ∆, absolute change from baseline to week 6; %∆, percent change from baseline to week 6; 7αOHCA; 7αOH-3-oxo-4-cholestenoic acid; ApoCA; apocholic acid; ASV, amplicon sequence variant; AUC, area under the curve; AX, arabinoxylan; BL, baseline; HDCA, hyodeoxycholic acid; HOMA-IR, homeostatic model assessment of insulin resistance; MCC, microcrystalline cellulose; OTU, operational taxonomic unit; TNF-α, tumor necrosis factor-α. [file 40168_2022_1248_MOESM12_ESM.pdf]
