# Supplementary material for: Elucidating the role of the gut microbiota in the physiological effects of dietary fiber
Source: Microbiome. 2022 May 13;10:77. doi: 10.1186/s40168-022-01248-5 (PMC9107176; doi:10.1186/s40168-022-01248-5)
Supplement: Supplementary file 3 — Additional file 2: Table S1. Baseline clinical measurements. Table provides the anthropometric measurements, surrogate endpoints, and biomarkers of host-microbiota interactions assessed at baseline, with participant grouped by arabinoxylan or microcrystalline cellulose supplementation. Data provided as mean ± SD. [file 40168_2022_1248_MOESM2_ESM.pdf]

Additional file 2: Table S1. Baseline Clinical Measurements.

	Arabinoxylan (*n*=15)	Microcrystalline Cellulose (*n*=16)	Between Group *p* value
Sex (F/M)	10/5	11/5	
Age (y)	33.7 ± 9.7	32.1 ± 7.4	1.00
**Anthropometric Measurements**			
Body weight (kg)	84.8 ± 12.3	81.9 ± 10.5	0.61
BMI (kg/m^2)	28.7 ± 2.7	28.7 ± 2.0	0.98
Waist circumference (cm)	95.7 ± 8.7	92.9 ± 6.0	0.34
Body fat % - females	36.4 ± 2.9	38.0 ± 6.1	0.42
Body fat % - males	22.5 ± 3.6	23.0 ± 5.3	0.87
**Surrogate Endpoints**			
Systolic (mm Hg)	118.5 ± 13.6	120.6 ± 15.7	0.59
Diastolic (mm Hg)	71.4 ± 9.0	74.9 ± 12.3	0.32
Pulse (beats/min)	73.1 ± 12.8	74.2 ± 12.6	0.84
Red blood cells (10^{12}/L)	4.7 ± 0.3	4.6 ± 0.5	0.44
Hemoglobin (g/L)	140.7 ± 11.6	134.2 ± 14.9	0.12
Hematocrit (L/L)	0.42 ± 0.03	0.40 ± 0.04	0.20
White blood cells (10^9/L)	6.3 ± 1.1	6.3 ± 1.5	1.00
Platelets (10^9/L)	243.8 ± 35.1	243.4 ±42.7	0.94
Glucose (mmol/L)	5.1 ± 0.4	5.0 ± 0.4	0.84
Insulin (pg/mL)	649.9 ± 253.5	593.7 ± 254.3	0.55
HOMA-IR	3.37 ± 1.34	3.02 ± 1.22	0.36
QUICKI	0.288 ± 0.2	0.292 ± 0.2	0.84
Total cholesterol (mmol/L)	4.4 ± 0.8	4.6 ± 1.0	0.57
LDL cholesterol (mmol/L)	2.5 ± 0.6	2.7 ± 0.7	0.94
HDL cholesterol (mmol/L)	1.3 ± 0.3	1.3 ± 0.3	1.00
Triglycerides (mmol/L)	1.2 ± 0.8	1.4 ± 0.7	0.96
hs-CRP (mg/L)	4.0 ± 4.6	3.6 ± 3.5 [b]	0.84
Fecal calprotectin (mg/kg)	66.4 ± 45.2	63.4 ± 53.7	0.90
**Biomarkers of Host-Microbiota Interactions**			
Glucagon (pg/mL)	137.0 ± 40.2	137.6 ± 61.0	0.98
GLP-1 (pg/mL)	13.9 ± 4.2	13.5 ± 6.3	0.76
PYY (pg/mL)	62.6 ± 25.4	48.7 ± 15.4	0.10
Ghrelin (pg/mL)	583.6 ± 291.0	687.4 ± 223.5	0.18
Leptin (ng/mL)	28.5 ± 25.0	30.2 ± 27.5 [a]	0.80
Adiponectin (mg/L)	15.0 ± 5.2	18.7 ± 10.9	0.22
TNF-α (pg/mL)	2.6 ± 0.6	2.7 ± 0.6	0.94
IL-6 (pg/mL)	0.69 ± 0.45	0.66 ± 0.25	0.98
IL-8 (pg/mL)	3.2 ± 1.0 [a]	3.3 ± 0.8	0.96
IL-10 (pg/mL)	0.25 ± 0.07	0.22 ± 0.07	0.28
LBP (µg/mL)	52.1 ± 17.9	53.9 ± 14.7	0.82
Fecal albumin (mg/L)	5.1 ± 6.8	3.1 ± 4.7	0.29
Fecal zonulin (µg/mL)	0.41 ± 0.44 [a]	0.21 ± 0.34	0.18
TMAO (µM)	3.7 ± 1.8	2.9 ± 1.3	0.19

Statistical significances of between-group differences at baseline (AX vs MCC) were determined by unpaired permutational *t*-tests. Data are means ± SD. Statistical significance was set at $p<0.01$.

[a] One outlier >5*SD from the mean was excluded; [b] Two outliers >5*SD from the mean were excluded.

Abbreviations: BMI, body mass index; GLP-1, active glucagon-like peptide-1; HDL, high-density lipoprotein; hs-CRP, high-sensitivity C-reactive protein; HOMA-IR, homeostatic model assessment of insulin resistance; IL, interleukin; LBP, lipopolysaccharide-binding protein; LDL, low-density lipoprotein; PYY, total peptide tyrosine tyrosine; QUICKI, quantitative insulin sensitivity check index; TMAO, trimethylamine *N*-oxide; TNF-α, tumor necrosis factor-α.
