# Supplementary material for: Elucidating the role of the gut microbiota in the physiological effects of dietary fiber
Source: Microbiome. 2022 May 13;10:77. doi: 10.1186/s40168-022-01248-5 (PMC9107176; doi:10.1186/s40168-022-01248-5)
Supplement: Supplementary file 4 — Additional file 3: Table S2. Macronutrient consumption at baseline and during arabinoxylan or microcrystalline cellulose supplementation. Table provides total dietary calories and macronutrients as assessed by the Canadian version of the web-based Automated Self-Administered 24-hour Dietary Assessment Tool, with participant grouped by arabinoxylan or microcrystalline cellulose supplementation. Data provided as mean ± SD. [file 40168_2022_1248_MOESM3_ESM.pdf]

Additional file 3: Table S2. Macronutrient Consumption at Baseline and During Arabinoxylan or Microcrystalline Cellulose Supplementation.

	Arabinoxylan (*n*=15)				Microcrystalline Cellulose (*n*=16)				Between
	Baseline	During Intervention	Within Group *p* value	Change (%)	Baseline	During Intervention	Within Group *p* value	Change (%)	Group Change *p* value
**Energy (kcal)**	2344 ± 1230	2331 ± 978	0.95	9 ± 38	1939 ± 505	2220 ± 648	0.06	17 ± 27	0.63
**Carbohydrates (g)**	262 ± 133	273 ± 130	0.66	11 ± 35	221 ± 76	257 ± 89	**0.046**	20 ± 31	0.21
Sugar (g)	114 ± 58	138 ± 72	**0.04**	35 ± 62	90 ± 35	118 ± 54	**0.03**	46 ± 80	0.55
Background dietary fiber (g) [a]	21 ± 6	19 ± 8	0.56	0.3 ± 46	19 ± 8	18 ± 5	0.41	3 ± 39	0.92
Background dietary fiber plus fiber supplement (g) [b]	21 ± 6	46 ± 12	**0.0002**	142 ± 84	19 ± 8	44 ± 8	**0.0002**	171 ± 113	0.50
Females (g) [b]	19 ± 4	40 ± 5	**0.0002**	128 ± 81	19 ± 6	40 ± 6	**0.0002**	140 ± 101	0.69
Males (g) [b]	24 ± 9	58 ± 13	**0.006**	169 ± 93	19 ± 13	53 ± 6	**0.0004**	239 ± 116	0.15
**Proteins (g)**	99 ± 62	97 ± 35	0.82	19 ± 67	81 ± 37	94 ± 25	0.17	26 ± 39	0.68
**Total fats (g)**	98 ± 60	96 ± 42	0.83	21 ± 68	76 ± 25	87 ± 33	0.15	19 ± 43	0.98
Saturated fat (g)	32 ± 23	32 ± 16	0.94	43 ± 126	26 ± 12	33 ± 14	0.09	37 ± 65	1.00
Unsaturated fat (g)	58 ± 34	56 ± 24	0.73	16 ± 61	43 ± 12	47 ± 17	0.26	11 ± 35	0.82
**Cholesterol (mg)**	358 ± 236	419 ± 183	**0.047**	56 ± 88	335 ± 166	376 ± 128	0.31	34 ± 68	0.46

Statistical significances of changes within-group were determined by paired permutational *t*-test, while between-group differences of percent change (AX vs MCC; during intervention – baseline/baseline*100) were determined by unpaired permutational *t*-test. Data are means ± SD. Statistical significance was set at $p<0.05$ (bolded *p* values).

[a] Total dietary fiber provided by the background diet without consideration of the supplemented fiber.

[b] Total dietary fiber provided by the background diet plus supplemental fiber when considering adherence to the intervention protocol.
