# Supplementary material for: Elucidating the role of the gut microbiota in the physiological effects of dietary fiber
Source: Microbiome. 2022 May 13;10:77. doi: 10.1186/s40168-022-01248-5 (PMC9107176; doi:10.1186/s40168-022-01248-5)
Supplement: Supplementary file 5 — Additional file 4: Table S3. Clinical measurements at baseline and six weeks of arabinoxylan or microcrystalline cellulose supplementation. Table provides the anthropometric measurements, surrogate endpoints, biomarkers of host-microbiota interactions, and stool characteristics assessed, with participant grouped by arabinoxylan or microcrystalline cellulose supplementation. Data provided as mean ± SD. [file 40168_2022_1248_MOESM4_ESM.pdf]

Additional file 4: Table S3. Clinical Measurements at Baseline and Six Weeks of Arabinoxylan or Microcrystalline Cellulose Supplementation.

	Arabinoxylan (*n*=15)				Microcrystalline Cellulose (*n*=16)				Between Group Change *p* value
	Baseline	Week 6	Within Group *p* value	Change (%)	Baseline	Week 6	Within Group *p* value	Change (%)	
**Anthropometric Measurements**									
Body weight (kg)	84.8±12.3	85.2±12.9	0.45	0.4±1.9	81.9±10.5	81.3±10.2	0.12	-0.8±1.9	0.17
BMI (kg/m^2)	28.7±2.7	28.8±2.8	0.47	0.4±1.9	28.7±2.0	28.5±2.1	0.13	-0.8±1.9	0.11
WC (cm)	95.7±8.7	95.5±8.1	0.81	-0.1±3.2	92.9±6.0	91.9±6.8	0.16	-1.2±3.0	0.43
Body fat % - females	36.4±2.9	36.6±3.6	0.22	0.7±3.9	38.0±6.1	38.5±6.1	0.50	1.5±3.4	0.82
Body fat % - males	22.5±3.6	23.0±3.2	0.61	2.3±4.1	23.0±5.3	23.2±5.6	0.30	1.1±4.3	1.00
**Surrogate Endpoints**									
Systolic (mm Hg)	118.5±13.6	116.6±13.6	0.28	-1.5±5.5	120.6±15.7	120.3±17.8	0.89	-0.2±6.4	0.60
Diastolic (mm Hg)	71.4±9.0	71.4±8.7	1.00	0.4±7.4	74.9±12.3	75.3±12.1	0.77	0.8±7.0	0.92
Pulse (beats/min)	73.1±12.8	70.6±10.3	0.15	-2.6±8.6	74.2±12.6	77.3±11.2	0.18	5.4±13.9	0.12
RBC (10^{12}/L)	4.7±0.3	4.7±0.3	0.77	0.6±5.1	4.6±0.5	4.7±0.5	**0.02**	2.4±3.0	0.27
Hemoglobin (g/L)	140.7±11.6	141.3±9.7	1.00	0.6±4.1	134.2±14.9	136.6±15.1	0.07	1.8±3.0	0.71
Hematocrit (L/L)	0.42±0.03	0.42±0.03	1.00	0.3±4.1	0.40±0.04	0.41±0.04	**0.03**	2.2±2.6	0.10
WBC (10^9/L)	6.3±1.1	6.2±1.1	0.75	0.1±13.4	6.3±1.5	6.3±1.2	1.00	2.6±16.6	0.58
Platelets (10^9/L)	243.8±35.1	251.9±61.6	1.00	2.8±17.3	243.4±42.7	253.8±51.0	0.23	4.4±10.6	0.82
Glucose (mmol/L)	5.1±0.4	5.0±0.4	0.21	-1.8±5.5	5.0±0.4	5.1±0.5	0.63	0.7±6.0	0.69
Insulin (pg/mL)	649.9±253.5	544.4±200.1	**0.046**	-10.2±36.3	593.7±254.3	658.7±177.5	0.21	22.5±38.1	**0.02**
HOMA-IR	3.4±1.3	2.8±1.2	**0.04**	-11.8±35.5	3.0±1.2	3.4±0.9	0.23	24.6±44.0	**0.006 ***
QUICKI	0.288±0.02	0.295±0.02	0.06	2.5±4.6	0.292±0.02	0.286±0.01	0.06	-2.0±4.4	**0.008 ***
Total-C (mmol/L)	4.4±0.8	4.6±0.8	**0.02**	4.9±7.1	4.6±1.0	4.7±1.0	0.42	2.2±9.9	0.43
LDL-C (mmol/L)	2.5±0.6	2.7±0.7	**0.03**	9.3±14.6	2.7±0.7	2.7±0.9	0.63	1.7±18.1	0.39
HDL-C (mmol/L)	1.3±0.3	1.3±0.3	0.65	2.8±12.9	1.3±0.3	1.2±0.3	0.12	-3.9±9.1	0.09
Triglycerides (mmol/L)	1.2±0.8	1.1±0.5	0.59	9.8±43.2	1.4±0.7	1.6±0.6	0.23	30.0±57.7	0.33
hs-CRP (mg/L)	4.0±4.6	3.1±3.2	0.25	5.7±66.1	3.6±3.5 [b]	3.5±3.4 [b]	0.71	-0.6±37.4 [b]	0.82
f-Calprotectin (mg/kg)	66.4±45.2	63.8±55.1	0.85	14.3±77.5	63.4±53.7	38.5±35.5	**0.004 ***	-39.1±24.0	**0.002 ***
**Biomarkers of Host-Microbiota Interactions**									
Glucagon (pg/mL)	137.0±40.2	129.6±34.6	0.09	-3.7±12.7	137.6±61.0	132.3±57.1	0.28	-2.9±11.6	0.88
GLP-1 (pg/mL)	13.9±4.2	12.3±5.5	**0.047**	-15.6±27.7	13.5±6.3	13.6±6.2	0.96	5.1±58.3	0.22
PYY (pg/mL)	62.6±25.4	56.9±21.8	0.13	-6.4±23.9	48.7±15.4	49.6±19.1	0.82	3.1±32.5	0.35
Ghrelin (pg/mL)	583.6±291.0	539.8±427.1	0.57	-7.8±33.9	687.4±223.5	670.7±262.6	0.65	-2.8±20.2	0.66
Leptin (ng/mL)	28.5±25.0	27.6±18.0	0.73	21.7±42.4	30.2±27.5 [a]	31.0±25.6 [a]	0.69	9.7±29.2 [a]	0.38
Adiponectin (mg/L)	15.0±5.2	16.6±6.7	0.10	11.1±19.0	18.7±10.9	18.6±11.4	0.93	1.7±14.8	0.12
TNF-α (pg/mL)	2.6±0.6	2.5±0.6	0.36	-2.0±13.6	2.7±0.6	2.5±0.6	**0.004 ***	-6.7±8.7	0.67
IL-6 (pg/mL)	0.69±0.45	0.67±0.36	0.86	11.0±49.0	0.66±0.25	0.61±0.27	0.32	-2.8±33.1	0.51
IL-8 (pg/mL)	3.2±1.0 [a]	3.2±1.2 [a]	0.91	0.7±19.8 [a]	3.3±0.8	3.4±0.7	0.52	4.8±18.0	0.64
IL-10 (pg/mL)	0.25±0.07	0.25±0.12	0.82	-1.0±34.9	0.22±0.07	0.24±0.07	0.23	12.7±30.9	0.15
LBP (µg/mL)	52.1±17.9	50.7±17.1	0.80	3.1±39.1	53.9±14.7	48.1±14.3	0.09	-7.6±26.7	0.76
f-Albumin (mg/L)	5.1±6.8	5.1±5.2	0.96	78.8±207.9	3.1±4.7	2.7±3.9	0.71	48.7±197.4	1.00
f-Zonulin (µg/mL)	0.41±0.44 [a]	0.43±0.60 [a]	0.85	48.9±116.1 [a]	0.21±0.34	0.25±0.43	0.42	28.2±103.4	0.80
TMAO (µM)	3.7±1.8	3.1±1.4	0.35	3.2±62.1	2.9±1.3	2.8±1.4	0.93	16.1±78.0	0.58
**Stool characteristics** [c]				**AUC$_{BL\text{-}W6}$** [c]				**AUC$_{BL\text{-}W6}$** [c]	
Stool consistency	1.9±1.1	2.3±0.5	0.65	12.9±3.3	1.8±0.9	1.8±0.9	1.00	10.9±2.2	0.06
Bowel frequency	1.7±0.8	2.1±0.7	0.47	13.0±4.5	2.1±0.9	2.1±1.0	1.00	13.3±5.4	0.81

Statistical significances of changes within-group were determined by paired permutational *t*-tests, while between-group differences (AX vs MCC; week 6 – baseline/baseline*100) were determined by unpaired permutational *t*-tests. Data are means ± SD.

* Statistical significance was set at $p<0.01$, bolded *p* values without an asterisk (*) are approaching statistical significance ($p<0.05$).

[a] One outlier >5*SD from the mean was excluded; [b] Two outliers >5*SD from the mean were excluded; [c] referred to our preceding study[1], changes within-group were determined by GEE models, while between-group differences in the area under the curve from baseline to week 6 (AUC$_{BL\text{-}W6}$) were determined by Mann-Whitney tests.

Abbreviations: BMI, body mass index; f-, quantified in feces; GLP-1, active glucagon-like peptide-1; HDL-C, high-density lipoprotein cholesterol; hs-CRP, high-sensitivity C-reactive protein; HOMA-IR, homeostatic model assessment of insulin resistance; IL, interleukin; LBP, lipopolysaccharide-binding protein; LDL-C, low-density lipoprotein cholesterol; PYY, total peptide tyrosine tyrosine; QUICKI, quantitative insulin sensitivity check index; Total-C, total cholesterol; TMAO, trimethylamine *N*-oxide; TNF-α, tumor necrosis factor-α; WC, waist circumference; WBC, white blood cells.
