# Supplementary material for: Elucidating the role of the gut microbiota in the physiological effects of dietary fiber
Source: Microbiome. 2022 May 13;10:77. doi: 10.1186/s40168-022-01248-5 (PMC9107176; doi:10.1186/s40168-022-01248-5)
Supplement: Supplementary file 6 — Additional file 5: Table S4. Covariate-adjustment of dietary fiber treatment effects by analysis of covariance (ANCOVA). Table provides p values from separate ANCOVA models used to adjust for the following covariates: age, sex, total dietary fiber intake, total dietary sugar intake, stool consistency, and bowel movement frequency. [file 40168_2022_1248_MOESM5_ESM.pdf]

Additional file 5: Table S4. Covariate-Adjustment of Dietary Fiber Treatment Effects by ANCOVA.

	Within AX Group (Baseline vs Week 6; *n*=15)						Within MCC Group (Baseline vs Week 6; *n*=16)						Between Group (AX vs MCC)					
	M1	M2	M3	M4	M5	M6	M1	M2	M3	M4	M5	M6	M1	M2	M3	M4	M5	M6
Satiety after a meal [a]	--	--	--	--	--	--	--	--	--	--	--	--	**0.03**	**0.04**	**0.03**	**0.03**	**0.04**	**0.04**
HOMA-IR [b]	**0.04**	**0.04**	0.33	0.18	0.11	**0.03**	0.23	0.23	0.06	0.57	0.21	0.22	**0.02**	**0.005**	**0.02**	**0.01**	**0.03**	**0.03**
QUICKI [b]	0.07	0.06	0.50	0.29	0.13	0.05	0.06	0.06	**0.01**	0.20	0.06	0.06	**0.005**	**0.005**	**0.01**	**0.005**	**0.006**	**0.01**
Fecal calprotectin [b]	0.85	0.85	0.18	0.96	0.85	0.94	**0.002**	**0.004**	0.98	**0.007**	**0.006**	**0.005**	**0.01**	**0.01**	**0.007**	**0.007**	**0.005**	**0.005**
Tumor necrosis factor-α [b]	0.37	0.36	0.06	0.21	0.38	0.18	**0.006**	**0.005**	**<0.001**	**0.007**	**0.03**	**0.006**	0.51	0.32	0.24	0.47	0.42	0.20

M1: model adjusted for age. **M2**: model adjusted for sex. **M3**: model adjusted for changes in total dietary fiber intake, which considers the amount of supplemental fiber. **M4**: model adjusted for changes in total dietary sugar intake. **M5**: model adjusted for differences in stool consistency. **M6**: model adjusted for differences in bowel movement frequency. Statistical significances of changes within-group were determined by repeated measures permutational ANCOVA models, while between-group differences of either ([a]) area under the curve or ([b]) percent change from baseline (during intervention – baseline/baseline*100) were determined by unpaired permutational ANCOVA. Data presented as *p* values with significance set at $p<0.05$ (bolded *p* values).

Abbreviations: AX, arabinoxylan; ANCOVA, analysis of covariance; HOMA-IR, homeostatic model assessment of insulin resistance; MCC, microcrystalline cellulose; QUICKI, quantitative insulin sensitivity check index.
