# Supplementary material for: Elucidating the role of the gut microbiota in the physiological effects of dietary fiber
Source: Microbiome. 2022 May 13;10:77. doi: 10.1186/s40168-022-01248-5 (PMC9107176; doi:10.1186/s40168-022-01248-5)
Supplement: Supplementary file 7 — Additional file 6: Table S5. Fecal concentrations of remaining bile acids detected at baseline and six weeks of arabinoxylan or microcrystalline cellulose supplementation. Table provides the remaining 29 bile acid compounds that were detected in < 90% of fecal samples, with participant grouped by arabinoxylan or microcrystalline cellulose supplementation. [file 40168_2022_1248_MOESM6_ESM.pdf]

Additional file 6: Table S5. Fecal Concentrations of Remaining Bile Acids Detected at Baseline and Six Weeks of Arabinoxylan or Microcrystalline Cellulose Supplementation.

Fecal Bile Acids (nmol/g)	Arabinoxylan (*n*=15)				Microcrystalline Cellulose (*n*=16)				Between Group Change *p* value
	Baseline	Week 6	Within Group *p* value	Change (W6-BL)	Baseline	Week 6	Within Group *p* value	Change (W6-BL)	
Tauro-α-muricholic acid	1.5±1.9	1.9±3.5	0.69	0.4±0.29	1.5±2.5	1.1±2.9	0.52	-0.4±2.2	0.38
α-Muricholic acid	0.81±1.52 [a]	0.12±0.44 [a]	0.18	-0.68±1.65 [a]	0.44±1.56	0.17±0.51	0.77	-0.26±1.69	0.53
β-Muricholic acid	0.49±1.36	0.11±0.26	0.45	-0.38±1.41	0.23±0.42 [a]	0.43±0.83 [a]	0.59	0.21±0.90 [a]	0.23
λ-Muricholic acid	7.5±15.3 [a]	2.6±3.9 [a]	0.36	-4.9±15.3 [a]	0.6±1.2	6.3±15.5	0.23	5.6±15.3	0.05
Ш-Muricholic acid	115.3±237.7 [a]	74.4±85.4 [a]	0.67	-40.9±226.0 [a]	28.9±55.0	174.1±439.6	0.41	145.2±433.2	0.12
Dehydrocholic acid	2.8±4.0	3.6±6.1	0.68	0.8±6.9	1.9±3.5	1.1±2.6	0.49	-0.8±4.0	0.31
Glycohyocholic acid	0.02±0.06 [a]	0.00±0.00 [a]	0.67	-0.02±0.06 [a]	0.02±0.08	0.03±0.12	0.75	0.01±0.15	0.69
Glycohyodeoxycholic acid	0.08±0.11	0.03±0.03	0.28	-0.05±0.12	0.09±0.14	0.04±0.08	0.06	-0.05±0.10	1.00
Glycoursodeoxycholic acid	2.1±3.3 [a]	1.8±2.1 [a]	0.61	-0.2±1.8 [a]	2.1±2.9	0.9±1.1	0.14	-1.2±2.6	0.78
Taurodeoxycholic acid	4.3±4.9	9.0±13.0	0.25	4.7±13.4	13.9±24.3 [a]	1.4±2.2 [a]	**0.04**	-12.5±23.8 [a]	**0.014**
Taurohyocholic acid	0.06±0.23 [a]	0.06±0.14 [a]	0.91	0.00±0.28 [a]	0.03±0.07	0.02±0.07	0.85	-0.01±0.10	0.88
Ursodeoxycholic acid	510.0±1084.6 [a]	147.1±209.7 [a]	0.28	-362.9±997.7 [a]	25.3±37.6	283.1±772.9	0.45	257.9±772.0	**0.017**
Norursodeoxycholic acid	0.63±0.83	0.43±0.58	0.52	-0.20±0.98	0.52±0.65	0.19±.041	0.11	-0.33±0.80	0.58
12-Ketolithocholic acid	0.46±1.03	0.44±0.87	0.87	-0.02±1.27	0.61±0.82	0.32±0.54	0.24	-0.29±0.88	0.64
6,7-diKetolithocholic acid	0.10±0.21	0.01±0.05	0.13	-0.09±0.18	0.45±0.73	0.23±0.33	0.30	-0.23±0.78	0.64
3-Oxocholic acid	1.4±4.6 [a]	1.4±2.9 [a]	0.92	0.0±4.3 [a]	0.1±0.2	3.9±10.5	0.27	3.8±10.5	0.27
diOxolithocholic acid	1.2±1.0	0.9±1.4	0.39	-0.3±1.8	0.88±0.84	0.42±0.55	0.10	-0.46±0.99	0.80
3β7α-diOH-5-cholestenoic acid	0.09±0.19	0.10±0.22	0.95	0.01±0.33	0.08±0.31 [a]	0.04±0.17 [a]	0.85	-0.04±0.36 [a]	0.76
Allocholic acid 3-SO_4^{2-}	68.6±177.8	50.2±140.0	0.84	-18.5±206.0	72.5±194.8	49.3±123.3	0.54	-23.2±118.9	0.94
Glycocholic acid 3-SO_4^{2-}	0.01±0.03	0.01±0.04	0.98	0.00±0.05	0.01±0.04	0.00±0.01	0.36	-0.01±0.04	0.67
Glycochenodeoxycholic acid 3-SO_4^{2-}	0.22±0.60 [a]	0.12±0.27 [a]	0.73	-0.10±0.70 [a]	0.27±0.51	0.12±0.25	0.25	-0.15±0.58	0.94
Glycoursodeoxycholic acid 3-SO_4^{2-}	0.16±0.34	0.04±0.08	0.22	-0.12±0.33	0.04±0.12	0.09±0.29	0.50	0.05±0.18	0.08
Taurolithocholic acid 3-SO_4^{2-}	1.5±1.1	0.7±0.9	0.06	-0.8±1.5	1.9±3.4 [a]	1.2±1.5 [a]	0.58	-0.8±3.8 [a]	1.00
Taurodeoxycholic acid 3-SO_4^{2-}	0.17±0.20	0.57±1.68	0.66	0.39±1.56	0.44±0.92 [a]	0.06±0.09 [a]	0.13	-0.39±0.94 [a]	0.06
Taurochenodeoxycholic acid 3-SO_4^{2-}	0.18±0.39	0.21±0.38	0.73	0.03±0.60	0.22±0.49	0.11±0.26	0.32	-0.12±0.54	0.82
Tauroursodeoxycholic acid 3-SO_4^{2-}	0.11±0.18	0.02±0.08	0.12	-0.08±0.21	0.05±0.13	0.02±0.08	0.46	-0.03±0.16	0.34
Chenodeoxycholic acid 3-glucuronide	0.18±0.46	0.17±0.44	0.98	-0.01±0.48	0.16±0.43	0.23±0.50	0.84	0.08±0.72	0.62
Chenodeoxycholic acid 24-glucuronide	0.06±0.12	0.08±0.14	0.76	0.02±0.18	0.09±0.14	0.05±0.11	0.45	-0.04±0.20	0.42
Lithocholic acid 24-glucuronide	0.00±0.00	0.09±0.19	0.13	0.09±0.19	0.07±0.19	0.05±0.18	0.86	-0.03±0.28	0.29

Listed bile acids (29 compounds) were detected in <90% of fecal samples. Statistical significances of within-group shifts were determined by paired permutational *t*-tests, while between-group differences (AX vs MCC; W6-BL, week 6 - baseline) were determined by unpaired permutational *t*-tests. Data are means ± SD. Statistical significance was set at *p*<0.01, bolded *p* values are approaching statistical significance (*p*<0.05). [a] One outlier >5*SD from the mean was excluded.
