# Supplementary material for: Elucidating the role of the gut microbiota in the physiological effects of dietary fiber
Source: Microbiome. 2022 May 13;10:77. doi: 10.1186/s40168-022-01248-5 (PMC9107176; doi:10.1186/s40168-022-01248-5)
Supplement: Supplementary file 9 — Additional file 8: Table S6. Relative abundance of bacterial taxa activated through ex vivo incubation with arabinoxylan and recovered by fluorescence-activated cell sorting (FACS). Table provides α-diversity and the bacterial taxa that were activated at 6-h incubation with arabinoxylan, recovered by FACS, showed a mean relative abundance > 1%, and/or showed a differential abundance in the recovered consortia relative to the total fecal communities. Data provided as mean ± SD. [file 40168_2022_1248_MOESM8_ESM.pdf]

Additional file 8: Table S6. Relative Abundance of Bacterial Taxa Activated through *ex vivo* Incubation with Arabinoxylan and Recovered by FACS.

α-Diversity Index or Taxonomic Group	Arabinoxylan (*n*=15)						
	Time 0	Time 6	FACS Sorted	FACS vs Time 0 Log_2 Fold Difference	FACS vs Time 0 *p* value	FACS vs Time 6 Log_2 Fold Difference	FACS vs Time 6 *p* value
**α-Diversity Index** [a]							
Shannon Index	3.60±0.26	3.66±0.23	2.75±0.89	-0.50	**0.0008**	-0.52	**0.002**
Inverse Simpson Index	20.35±8.61	22.09±7.99	11.82±9.13	-1.15	**0.0004**	-1.30	**0.0002**
Chao1 Index	215.59±48.53	210.46±40.21	159.06±23.88	-0.42	**<0.0001**	-0.39	**<0.0001**
Total Number of ASVs	167.73±39.9	168.00±32.22	115.27±17.97	-0.51	**0.0001**	-0.53	**0.0001**
**Phyla** [b]							
Bacteroidetes	42.25±7.40	41.69±8.77	43.83±27.84	0.41	0.09	0.37	0.13
Firmicutes	49.92±5.93	47.19±8.02	38.88±26.57	0.14	0.60	0.16	0.54
Proteobacteria	1.45±1.50	4.78±5.90	15.70±28.72	2.07	**0.001**	0.79	0.20
Actinobacteria	4.65±4.56	4.37±3.96	2.44±2.90	-0.60	0.34	-0.53	0.41
**Family** [b]							
Bacteroidaceae	28.27±14.89	31.40±13.34	39.58±27.12	1.55	**0.0006**	1.42	**0.001**
Lachnospiraceae	19.02±2.60	19.90±4.30	26.27±23.73	0.59	0.02	0.68	0.01
Enterobacteriaceae	0.83±1.31	4.36±5.89	15.57±28.71	5.34	**<0.0001**	3.48	**<0.0001**
Ruminococcaceae	12.64±5.05	11.32±5.60	5.43±6.74	-0.85	**0.001**	-0.62	0.05
Prevotellaceae	8.68±13.64	4.92±7.74	3.17±7.60	-1.36	0.26	-0.09	0.94
Bifidobacteriaceae	4.10±4.14	3.99±3.62	2.31±2.74	-0.44	0.50	-0.36	0.59
Veillonellaceae	7.76±9.51	5.63±6.04	1.47±1.84	-1.81	0.01	-1.14	0.10
Lactobacillaceae	0.03±0.06	0.09±0.14	1.02±1.42	6.54	**<0.0001**	5.38	**<0.0001**
Rikenellaceae	3.09±4.56	3.09±4.82	0.34±0.45	-2.67	**0.0001**	-2.75	**0.0003**
Streptococcaceae	1.88±1.74	1.97±2.31	0.30±0.26	-1.81	**0.002**	-1.78	**0.008**
**Genera** [b]							
Bacteroides	28.27±14.89	31.40±13.34	39.58±27.12	0.91	0.03	0.93	0.04
Escherichia/Shigella	0.83±1.31	4.36±5.89	15.57±28.71	3.13	**<0.0001**	3.04	**0.0005**
Blautia	3.75±2.06	4.20±1.24	10.40±16.22	0.63	0.04	0.66	0.05
Dorea	1.47±0.47	1.59±0.70	4.90±10.31	0.51	0.19	0.80	0.04
Lachnospiracea incertae sedis	3.08±2.53	3.41±2.50	2.68±2.71	-0.44	0.27	-0.27	0.51
Bifidobacterium	4.10±4.14	3.99±3.62	2.31±2.74	-0.76	0.24	-0.54	0.44
Roseburia	3.01±2.18	3.29±1.99	2.16±2.84	-1.23	**0.002**	-0.14	0.79
Paraprevotella	0.97±3.44	0.97±3.67	1.93±7.03	0.28	0.88	1.43	0.54
Fusicatenibacter	2.81±1.56	2.62±1.43	1.87±1.45	-0.76	0.09	-0.31	0.48
Ruminococcus	1.74±2.13	2.04±2.73	1.38±2.94	-0.75	0.31	-0.74	0.35
Faecalibacterium	5.34±2.99	4.38±2.62	1.34±1.58	-2.21	**<0.0001**	-1.55	**0.001**
Coprococcus	1.45±1.44	1.56±1.54	1.24±1.79	-0.53	0.31	-0.42	0.47
Prevotella	6.97±12.33	3.71±7.08	1.24±3.66	-3.35	0.04	-1.35	0.36
Clostridium XlVa	1.30±0.92	1.09±0.76	1.20±2.88	-0.36	0.58	-0.83	0.11
Gemmiger	1.22±1.06	1.00±0.80	1.06±1.22	0.06	0.93	0.61	0.35
Lactobacillus	0.03±0.06	0.09±0.14	1.02±1.42	5.90	**<0.0001**	4.96	**<0.0001**
Oscillibacter	1.61±0.97	1.55±1.11	0.47±0.57	-1.75	**0.0001**	-1.49	**0.003**
Alistipes	3.09±4.56	3.09±4.82	0.34±0.45	-3.20	**<0.0001**	-3.12	**<0.0001**
Streptococcus	1.57±1.11	1.83±2.28	0.30±0.26	-2.16	**0.003**	-1.97	**0.004**
Clostridium XVIII	0.75±0.53	0.67±0.55	0.15±0.18	-1.90	**0.008**	-1.38	0.06

Additional file 8: Table S6. Continued

α-Diversity Index or Taxonomic Group	Arabinoxylan (*n*=15)						
	Time 0	Time 6	FACS Sorted	FACS vs Time 0 Log_2 Fold Difference	FACS vs Time 0 *p* value	FACS vs Time 6 Log_2 Fold Difference	FACS vs Time 6 *p* value
**Amplicon sequence variant (ASV Number, closest hit in database, % identity)** [b]							
3ur8h3, *Escherichia fergusonii/Shigella sonnei*, 100%	0.90±1.55	4.50±6.39	15.59±28.82	3.20	**<0.0001**	1.19	0.10
2uaio6, *Bacteroides vulgatus*, 100%	3.55±3.52	4.14±4.51	4.96±6.85	0.54	0.48	1.06	0.13
4c03f6, *Bacteroides xylanisolvens*, 100%	0.61±1.10	1.44±2.58	4.82±9.40	2.96	**0.003**	2.06	0.04
fepklg, *Dorea longicatena*, 100%	1.16±0.49	1.38±0.49	4.49±10.19	-0.10	0.74	0.40	0.26
6eanz7, *Blautia massiliensis*, 100%	1.24±0.87	1.50±1.00	4.20±10.41	-0.25	0.46	-0.03	0.94
6wurws, *Bacteroides ovatus*, 100%	0.98±1.83	1.57±2.22	3.77±5.65	1.54	0.05	1.52	0.11
935uue *Bacteroides cellulosilyticus*, 100%	2.20±5.92	2.45±5.97	3.74±10.78	-0.52	0.64	-0.17	0.88
ff0431, *Bacteroides xylanisolvens*, 99.7%	0.36±0.66	0.91±1.76	3.14±6.70	2.49	0.02	1.65	0.12
tmdvof, *Bacteroides plebeius*, 100%	3.74±9.70	2.71±7.32	3.07±8.09	3.31	**0.001**	1.28	0.08
hcf4f5, *Blautia obeum*, 100%	1.09±1.16	0.81±0.64	2.01±3.63	-0.27	0.66	1.13	0.06
2st345, *Blautia faecis*, 100%	0.45±0.33	0.53±0.45	1.90±2.58	1.10	0.04	1.41	0.01
rfal2b, *Fusicatenibacter saccharivorans*, 100%	2.88±1.48	2.49±1.34	1.90±1.50	-1.40	**<0.001**	-0.37	0.27
e9ptub, *Blautia wexlerae*, 100%	0.86±0.66	0.96±0.56	1.89±2.30	0.62	0.13	0.91	0.05
5mi71s, *Bacteroides koreensis*, 99.7%	0.19±0.36	0.40±0.87	1.88±4.17	3.68	**0.0007**	3.31	**0.005**
4ma8bd, *Eubacterium rectale*, 100%	1.93±2.10	2.12±2.23	1.71±2.55	-0.75	0.22	-0.15	0.83
cbl07c, *Bacteroides ovatus*, 99.7%	0.31±0.47	0.55±0.76	1.66±3.19	1.85	0.07	1.93	0.08
jbtn3b, *Lachnospiraceae* sp.,	1.58±2.44	1.92±2.63	1.40±1.74	-0.96	0.28	-0.17	0.83
352ie0, *Bacteroides koreensis*, 99.7%	0.36±0.68	0.50±0.86	1.25±2.40	1.74	0.09	0.73	0.47
t9mxq7, *Bacteroides vulgatus*, 99.7%	1.46±1.80	1.69±2.60	1.20±2.44	-1.47	0.05	-0.57	0.46
ic9xvj, *Bifidobacterium longum*, 100%	2.10±2.48	2.17±1.99	1.11±1.53	-1.27	0.08	-0.91	0.23
hiw9kp, *Bacteroides dorei*, 100%	1.46±3.02	1.63±2.92	1.03±1.61	-0.73	0.33	-0.49	0.55
p3xfey, *Paraprevotella clara*, 97.8%	0.60±2.07	0.50±1.90	1.03±3.71	-0.87	0.51	2.54	0.16
pgue8q, *Lactobacillus crispatus/gallinarum*, 100%	0.02±0.05	0.09±0.14	0.97±1.41	7.03	**<0.0001**	5.10	**<0.0001**
49kgjb, *Ruminococcaceae*	0.87±1.09	0.75±0.97	0.43±1.17	-2.26	**0.0098**	-1.28	0.17
eb999b, *Faecalibacterium prausnitzii*, 99.7%	1.35±1.48	1.22±1.19	0.47±0.78	-2.20	**0.001**	-1.21	0.10
3rw028, *Faecalibacterium prausnitzii*, 98.8%	0.99±0.61	1.02±1.08	0.27±0.34	-2.40	**<0.0001**	-1.74	**0.006**
6pygnt, *Dialister invisus*, 100%	1.64±2.72	1.27±1.85	0.26±0.44	-2.80	**0.0007**	-2.35	**0.006**
bdmzc3, *Faecalibacterium prausnitzii*, 99%	1.44±1.70	0.76±0.80	0.21±0.36	-3.66	**<0.0001**	-1.98	**0.002**
19n89a, *Coprococcus eutactus*, 99.8%	0.28±0.64	0.26±0.67	0.18±0.66	-3.85	**0.001**	-2.62	0.12
gh9q6u, *Streptococcus thermophiles*, 100%	0.83±0.68	1.14±1.64	0.18±0.19	-2.52	**<0.0001**	-2.29	**0.0005**
dx5pax, *Faecalibacillus intestinalis*, 100%	0.82±0.56	0.72±0.58	0.17±0.16	-1.76	**0.006**	-1.03	0.12

Statistically significant differences between the bacterial consortia activated at 6-hours incubation with arabinoxylan and recovered by fluorescence-activated cell sorting (FACS) and the fecal bacterial community at 0-hours and 6-hours incubation with arabinoxylan were determined by either ([a]) one-way ANOVA with permutations or ([b]) testing for differences in the differential abundance of taxa using the R package DEseq2. Statistical significance was set at $p<0.01$ (bolded *p* values). Data are presented as mean relative abundance ± SD.
