# Supplementary material for: Elucidating the role of the gut microbiota in the physiological effects of dietary fiber
Source: Microbiome. 2022 May 13;10:77. doi: 10.1186/s40168-022-01248-5 (PMC9107176; doi:10.1186/s40168-022-01248-5)
Supplement: Supplementary file 11 — Additional file 10: Table S7. Univariate and covariate-adjusted generalized linear models (GLMs) assessing fecal microbiota-related factors that associate with surrogate endpoints of dietary fiber supplementation. Table provides β-coefficient directionality and p values from separate GLMs used to adjust for the following covariates: age, sex, total dietary fiber intake, total dietary sugar intake, stool consistency, and bowel movement frequency. [file 40168_2022_1248_MOESM10_ESM.pdf]

Additional file 10: Table S7. Univariate and Covariate-Adjusted GLMs Assessing Fecal Microbiota-Related Factors that Associate with Surrogate Endpoints of Dietary Fiber Supplementation.

	Within AX Group (*n*=15)							Within MCC Group (*n*=16)						
	M1	M2	M3	M4	M5	M6	M7	M1	M2	M3	M4	M5	M6	M7
**Satiety After a Meal (AUC)** [a]														
ASV6pygnt_*Dialister invisus* (%) [3]	↑ **0.03**	↑ **0.04**	↑ **0.03**	↑ **0.01**	↑ **0.02**	↑ **0.03**	↑ **0.03**	--	--	--	--	--	--	--
ASV2xmw96_*Dorea formicigenerans* (%) [3]	↓ **<0.001**	↓ **<0.001**	↓ **<0.001**	↓ **<0.001**	↓ **<0.001**	↓ **<0.001**	↓ **<0.001**	--	--	--	--	--	--	--
ASVeb999b_*Faecalibacterium prausnitzii* (%) [3]	↓ **0.03**	↓ **0.02**	↓ **0.03**	↓ 0.07	↓ 0.06	↓ **0.02**	↓ **0.04**	--	--	--	--	--	--	--
ASV56kx74_*Eubacterium ramulus* (%) [3]	↓ **0.01**	↓ **0.01**	↓ **0.02**	↓ **0.03**	↓ **0.02**	↓ **0.006**	↓ **0.02**	--	--	--	--	--	--	--
**HOMA-IR (%Δ)** [b]														
Lithocholic acid (Δ)	↑ **0.02**	↑ **0.03**	↑ **0.02**	↑ **0.01**	↑ **0.02**	↑ **0.04**	↑ **0.02**	↑ 0.84	↑ 0.85	↑ 0.76	↑ 0.83	↑ 0.96	↑ 0.80	↑ 0.89
Isolithocholic acid (Δ)	↑ **0.046**	↑ 0.09	↑ 0.05	↑ **0.049**	↑ **0.046**	↑ 0.06	↑ 0.05	↑ 0.53	↑ 0.53	↑ 0.45	↑ 0.49	↑ 0.57	↑ 0.41	↑ 0.56
**Fecal Calprotectin (%Δ)** [c]														
Taurolithocholic acid (Δ) [3]	↓ 0.15	↓ 0.17	↓ 0.15	↓ 0.22	↓ 0.27	↓ 0.22	↓ 0.09	↓ **0.001**	↓ **0.001**	↓ **0.001**	↓ **<0.001**	↓ **0.001**	↓ **<0.001**	↓ **<0.001**
Taurodeoxycholic acid (Δ) [3]	↓ 0.41	↓ 0.53	↓ 0.41	↓ 0.31	↓ 0.43	↓ 0.54	↓ 0.16	↓ **0.004**	↓ **0.005**	↓ **0.002**	**0.001**	↓ **0.004**	↓ **0.002**	↓ **0.001**
Glycodeoxycholic acid (Δ)	↑ 0.18	↑ 0.16	↑ 0.20	↑ 0.31	↑ 0.16	↑ 0.55	↑ 0.29	↓ **0.002**	↓ **0.002**	↓ **0.003**	**0.003**	↓ **0.001**	↓ **0.003**	↓ **<0.001**

[a] Satiety after a meal ($AUC_{BL\text{-}W6}$) was the dependent variable in Gaussian-distributed GLM with an identity link and the relative abundance of fluorescence-activated cell sorting (FACS) sorted amplicon sequence variants (ASV) as predictors.

[b] HOMA-IR (percent change) was the dependent variable in binomial-distributed GLM with a probit link and shifts in the concentration of fecal bile acids (absolute change) as predictors.

[c] Fecal calprotectin (percent change) was the dependent variable in Gaussian-distributed GLM with an identity link and shifts in the concentration of fecal bile acids (absolute change) as predictors.

M1: univariate model without adjustment. **M2**: model adjusted for age. **M3**: model adjusted for sex. **M4**: model adjusted for changes in total dietary fiber intake, which considers the amount of supplemental fiber (W6-BL). **M5**: model adjusted for changes in total dietary sugar intake (W6-BL). **M6**: model adjusted for differences in stool consistency during the intervention ($AUC_{BL\text{-}W6}$). **M7**: model adjusted for differences in bowel movement frequency during the intervention ($AUC_{BL\text{-}W6}$). Data presented as β-coefficient directionality plus *p* values (significance set at $p<0.05$, bolded *p* values).

Abbreviations: [3], cube root transformed prior to analysis; ASV, amplicon sequence variant; AX, arabinoxylan; AUC, area under the curve; BL, baseline; GLM, generalized linear model; HOMA-IR, homeostatic model assessment of insulin resistance; MCC, microcrystalline cellulose; W6, week 6.
