# Supplementary material for: Elucidating the role of the gut microbiota in the physiological effects of dietary fiber
Source: Microbiome. 2022 May 13;10:77. doi: 10.1186/s40168-022-01248-5 (PMC9107176; doi:10.1186/s40168-022-01248-5)
Supplement: Supplementary file 14 — Additional file 13: Table S8. Description of predictor datasets used for training the independent random forest classifiers. Table provides both a brief and detailed description of the variables included in each predictor dataset used for the random forest analyses, as referenced in Fig. 4 and Additional file 9: Figure S3. [file 40168_2022_1248_MOESM13_ESM.pdf]

Additional file 13: Table S8. Description of Predictor Datasets used for Training the Independent Random Forest Classifiers.

Brief Description	Detailed Description	Reference
%Δ Biomarkers	Percentage change from baseline to week 6 in the 14 biomarkers of host-microbiota interactions hypothesized to underlie the pathophysiology of obesity.	Table S3
Δ W1-BL SCFAs	Shorter-term shifts from baseline to week 1 in the fecal concentrations of acetate, propionate, butyrate, valerate, isobutyrate, and isovalerate; the relative proportions of acetate, propionate, and butyrate; and the propionate to butyrate ratio.	Nguyen *et al*. [1]
Δ W6-BL SCFAs	Longer-term shifts from baseline to week 6 in the fecal concentrations of acetate, propionate, butyrate, valerate, isobutyrate, and isovalerate; the relative proportions of acetate, propionate, and butyrate; and the propionate to butyrate ratio.	Nguyen *et al*. [1]
Δ All Bile Acids	Shifts from baseline to week 6 in all 60 bile acids detected.	Tables 1 and S5
Δ Prevalent Bile Acids	Shifts from baseline to week 6 in the 31 bile acids most frequently detected among fecal samples.	Table 1
Δ Responsive Bile Acids	Shifts from baseline to week 6 in the 14 bile acids that showed a tendency to respond to MCC or AX supplementation when compared to baseline ($p<0.1$, paired permutational t-tests).	Tables 1 and S5
Δ Ecological Features	Shifts in fecal α-diversity, intra-individual β-diversity, and the seven co-abundance response groups, plus *ex vivo* α-diversity and the principal components (PC1, PC2, and PC3) generated from the week 6 shifts of the 100 bacterial OTUs and the *ex vivo* relative abundance of the 90 bacterial ASVs.	Table S6 and Nguyen *et al*. [1]
BL Ecological Features	Baseline fecal α-diversity, baseline relative abundance of the seven co-abundance response groups, and the principal components (PC1, PC2, and PC3) generated from the 100 bacterial OTUs at baseline.	Nguyen *et al*. [1]
Δ Responsive OTUs	Shifts in the fecal relative abundance of the 18 bacterial OTUs that significantly changed by week 6 of AX consumption when compared to baseline or MCC ($q<0.15$, Wilcoxon or Mann–Whitney tests).	Nguyen *et al*. [1]
BL Responsive OTUs	Baseline fecal relative abundance of the 18 bacterial OTUs that significantly changed by week 6 of AX consumption when compared to baseline or MCC ($q<0.15$, Wilcoxon or Mann–Whitney tests).	Nguyen *et al*. [1]
Δ All OTUs	Shifts in the fecal relative abundance of the 100 bacterial OTUs with an average relative abundance ≥0.15%.	Nguyen *et al*. [1]
BL All OTUs	Baseline fecal relative abundance of the 100 bacterial OTUs with an average relative abundance ≥0.15%.	Nguyen *et al*. [1]
Diff. Abundant Metabolically Active ASVs	Relative abundance of the 14 bacterial ASVs shown to be differentially abundant in the BONCAT-labeled, FACS-recovered consortia at 6-hours incubation with AX when compared to the total fecal bacterial community at 0-hours incubation ($q<0.05$, DESeq2).	Table S6
All Metabolically Active ASVs	Relative abundance of the 90 bacterial ASVs shown to have an average relative abundance ≥0.15% among the BONCAT-labeled, FACS-recovered consortia detected at 6-hours incubation with AX.	Table S6
Δ Macronutrients	Change from BL to W6 in the calorie-adjusted intake of macronutrients assessed by ASA24-Canada-2014 (total carbohydrates, sugar, dietary fiber, protein, total fat, saturated fat, unsaturated fat, and cholesterol).	Table S2
BL Macronutrients	Baseline calorie-adjusted intake of macronutrients assessed by ASA24-Canada-2014 (total carbohydrates, sugar, dietary fiber, protein, total fat, saturated fat, unsaturated fat, and cholesterol).	Table S2

[1] Nguyen NK, Deehan EC, Zhang Z, Jin M, Baskota N, Perez-Muñoz ME, Cole J, Tuncil YE, Seethaler B, Wang T *et al*. Gut microbiota modulation with long-chain corn bran arabinoxylan in adults with overweight and obesity is linked to an individualized temporal increase in fecal propionate. Microbiome. 2020; 8:118.

Abbreviations: Δ, absolute change from baseline to week 6; %Δ, percent change from baseline to week 6; ASA24-Canada-2014, Canadian version of the web-based Automated Self-Administered 24-hour Dietary Assessment Tool; ASV, amplicon sequence variant; AX, arabinoxylan; BL, baseline; BONCAT, bioorthogonal non-canonical amino acid tagging; FACS, fluorescence-activated cell sorting; MCC, microcrystalline cellulose; OTU, operational taxonomic unit; SCFA, short-chain fatty acids; W6, week 6.
